# Daily handover in surgery: systematic review and a novel taxonomy of interventions and outcomes

**DOI:** 10.1093/bjsopen/zrae011

**Published:** 2024-03-01

**Authors:** Jessica M Ryan, Fiachra McHugh, Anastasija Simiceva, Walter Eppich, Dara O Kavanagh, Deborah A McNamara

**Affiliations:** RCSI SIM Centre for Simulation Education and Research, RCSI, Dublin, Ireland; StAR MD Programme, School of Postgraduate Studies, RCSI, Dublin, Ireland; Department of Surgery, The Bon Secours Hospital, Glasnevin, Dublin, Ireland; Department of Surgery, Mayo University Hospital, Mayo, Ireland; RCSI SIM Centre for Simulation Education and Research, RCSI, Dublin, Ireland; RCSI SIM Centre for Simulation Education and Research, RCSI, Dublin, Ireland; Department of Surgical Affairs, RCSI, Dublin, Ireland; Department of Surgery, Tallaght University Hospital, Dublin, Ireland; Office of the President, RCSI, Dublin, Ireland; National Clinical Programme in Surgery, RCSI, Dublin, Ireland; Department of Surgery, Beaumont Hospital, Dublin, Ireland

## Abstract

**Background:**

Poor-quality handovers lead to adverse outcomes for patients; however, there is a lack of evidence to support safe surgical handovers. This systematic review aims to summarize the interventions available to improve end-of-shift surgical handover. A novel taxonomy of interventions and outcomes and a modified quality assessment tool are also described.

**Methods:**

Ovid MEDLINE®, PubMed, Embase, and Cochrane databases were searched for articles up to April 2023. Comparative studies describing interventions for daily in-hospital surgical handovers between doctors were included. Studies were grouped according to their interventions and outcomes.

**Results:**

In total, 6139 citations were retrieved, and 41 studies met the inclusion criteria. The total patient sample sizes in the control and intervention groups were 11 946 and 11 563 patients, respectively. Most studies were pre-/post-intervention cohort studies (92.7%), and most (73.2%) represented level V evidence. The mean quality assessment score was 53.4% (17.1). A taxonomy of handover interventions and outcomes was developed, with interventions including handover tools, process standardization measures, staff education, and the use of mnemonics. More than 25% of studies used a document as the only intervention. Overall, 55 discrete outcomes were assessed in four categories including process (*n* = 27), staff (*n* = 14), patient (*n* = 12) and system-level (*n* = 2) outcomes. Significant improvements were seen in 51.8%, 78.5%, 58.3% (*n* = 9761 *versus* 9312 patients) and 100% of these outcomes, respectively.

**Conclusions:**

Most publications demonstrate that good-quality surgical handover improves outcomes and many interventions appear to be effective; however, studies are methodologically heterogeneous. These novel taxonomies and quality assessment tool will help standardize future studies.

## Teaser

This systematic review aimed to summarize the interventions available to improve end-of-shift surgical handover. A novel taxonomy of interventions and outcomes and modified quality assessment tool were also described.

Of the 6139 screened citations, 41 studies of 23 509 patients were identified, which evaluated the impact of four categories of handover interventions on 55 discrete outcomes. Studies were heterogeneous, and effective interventions included implementing a formal face-to-face handover meeting, an automated electronic handover document, a method to highlight critically unwell patients, ensuring appropriate senior supervision, staff education, and the use of a mnemonic or memory aid to structure patient presentations.

Future studies should prioritize these interventions and their effect on patient outcomes, particularly adverse events, ward round reviews and length of stay. The novel taxonomies described also provide a new language with which to describe handover research and create uniformity in future research studies.

## Introduction

Communication failures in healthcare are common, contribute significantly to adverse patient events and errors^1,2^ and cost an estimated $12 billion per year in U.S. hospitals^3^. An important communication event in the patient journey is the handover of care, which refers to ‘the exchange between health professionals of information about a patient accompanying either a transfer of control over, or of responsibility for, the patient’^4^.

One-quarter of handovers are associated with handover-related care failures^5^, and there are 7.5 handover-related issues with patient care per 100 patient days in hospital, mostly arising from omissions of critical information^6^. Surgical patients are particularly at risk, seeing an average of 10 different doctors during a single admission^7^, with changing work practices requiring information handover even more frequently due to shift changes. Shorter hospital stays among surgical patients increase the intensity of care and volume of clinical information. In one month, surgical interns participate in an average of 300 handovers, and in three days, each surgical patient will be handed over an average of 15 times^8^. Handovers are costly when they are not performed well, with one study extrapolating cost savings of between £740 000 and £3.82 million in one hospital with an improved surgical handover process^9^. Malpractice claims associated with communication failures are significantly more expensive to defend, and 40% of these claims are due to failed handovers^10^. Errors during information transfer also lead to wasted staff time^11^ and good-quality handover can reduce staff overtime^12^. The cost associated with this essential event to patients, staff and institutions means that an ad-hoc approach to handover improvement is not appropriate and changes should be supported by evidence.

Medical associations^13^, surgical colleges^14,15^ and the World Health Organization^16^ all offer guidance, but there is little evidence-based training for safe and effective surgical handover and no gold standard exists^15^. Handover interventions from other specialties are not always adaptable to the surgical ecosystem and are sometimes not rigorously evaluated prior to implementation^17^. In a review of articles published up to December 2013, only eight studies were found to address the daily surgical handover, with the majority focusing on the use of paper or electronic documents as interventions rather than overall process improvements^18^. Furthermore, the descriptions of methodologies employed were limited. The literature has increased significantly in the last 10 years; however, there is still little consensus on areas requiring further study.

The aim of this systematic review was to summarize and evaluate the literature on interventions used to improve the daily end-of-shift surgical handover. The authors sought to determine the types of interventions used, the outcome measures against which they were evaluated, and to assess the impact of interventions on outcome measures for surgical patients. Novel taxonomies have also been developed and reported for handover interventions and outcomes, and a modified quality assessment tool for handover research has been described.

## Methods

### Search strategy

This systematic review was prospectively registered on PROSPERO (CRD42022363198) and review methods were established prior to the conduct of this review. This review was also conducted in accordance with the PRISMA^19^ and AMSTAR (Assessing the Methodological Quality of Systematic Reviews) Guidelines^20^. PubMed, PubMed Central, Embase and Cochrane databases were searched for all articles published from inception until April 2023 using a search strategy developed with an Information Specialist (full search terms are in *Appendix S1*). The results were combined into a reference manager database (Endnote X20, Clarivate PLC, Jersey). Duplicates were removed automatically and manually. The reference lists of the included studies, prior reviews of the same or similar topics and the trial registry Clinicaltrials.gov were screened for additional relevant studies.

Original studies were included if they utilized any intervention to improve daily handover between surgical doctors and reported any outcomes related to the surgical handover process. All interventional study designs were included due to the small number of RCTs available. During the full-text review, studies involving students and newly appointed doctors who had not yet entered clinical practice were excluded, as a review of educational handover interventions was previously performed^21^. The full inclusion and exclusion criteria are listed in *Table 1*.

**Table 1:** Inclusion and exclusion criteria

PICO	Inclusion criteria	Exclusion criteria
**Population/setting**	Handover setting: In-hospital daily handovers between surgical doctors, including simulated handovers Staff: Surgical doctors from all specialties who participate in handover Patients: Patients admitted to any in-hospital surgical service	Nursing handover Handovers to non-surgical staff Studies involving medical students or those who have completed medical school but have not yet begun working in a clinical setting Out-of-hospital/inter-hospital handovers
**Intervention**	Any formal handover intervention	None
**Comparison**	Routine practice Informal handover No handover	None
**Outcomes**	Any	None

### Study screening and selection

Reviewers JR and FMcH independently applied inclusion and exclusion criteria to citations and abstracts to identify full texts for review. Full texts were then reviewed independently by both reviewers, with discrepancies agreed upon by consensus among the research team.

### Data extraction

A template was created using Microsoft Excel (16.67, ©2022 Microsoft) and a subset of papers was allocated to two reviewers (JR and FMcH) for independent primary data extraction, with subsequent validation of all papers by the second reviewer. Any discrepancies were resolved by consensus with the wider research team. Where a study was described in limited detail, reviewers contacted the authors for further details. For each study, data on the study characteristics, interventions, controls, outcomes and results were extracted.

### Data synthesis

Studies were first categorized according to the type of intervention used and then according to the outcomes assessed. A meta-analysis on this topic was not planned because of the high likelihood of clinical and methodological heterogeneity among the included studies^22^.

### Quality assessment

The authors planned to use multiple quality assessment (QA) tools for the various study types; however, a literature review revealed that a tool specifically designed to assess handover research existed^23^, which was based on a checklist designed to assess studies of randomized and non-randomized healthcare interventions from Downs and Black^24–26^.

This handover QA tool was noted by the authors to omit key characteristics of study quality, including internal validity, quality of reporting and power calculations, leading to the design of a modified tool to address these deficiencies (*Table S1*). Changes were based on the original checklist from Downs and Black^24^ and more recent commentary on improvement^17,27^. Items that had initially been excluded^23^ from the Downs and Black^24^ checklist were assessed by the study team and re-inserted into the modified tool if they addressed the above omitted characteristics. Both versions of the handover QA tool were used for all included studies^9,12,28–67^ and scores are reported in parallel. Two authors (FMcH and AS) independently performed QA for a subset of articles, while a third author (JR) reassessed and validated the QA scores for all articles. Outcomes were compared, with any discrepancies agreed upon by consensus.

### Development of intervention and outcome taxonomy

Categories of handover interventions were developed by the study team using deductive reasoning through a review of all included studies and previous similar reviews. For outcomes, the system described by Arora *et al.*^68^ was updated to include an additional category. Outcome subcategories were then added through deductive reasoning by the study team, ensuring all outcomes included in the current study were represented.

### Statistics

Data were analysed using Stata (17.0 ©2021, StataCorp, TX, USA). Descriptive data are presented as absolute values and percentages, and continuous data are presented as means and standard deviations (mean(s.d.)), and medians and ranges. Normality of QA scores was assessed using the Shapiro–Wilk test. Comparative analyses of quantitative data (QA scores and differences in study types across countries) were performed using the chi-square test with Yate’s correction for categorical variables and Student’s *t*-test for continuous variables. All tests of significance were two-tailed, with *P* < 0.05 indicating statistical significance.

## Results

### Search results

**Fig. 1:**
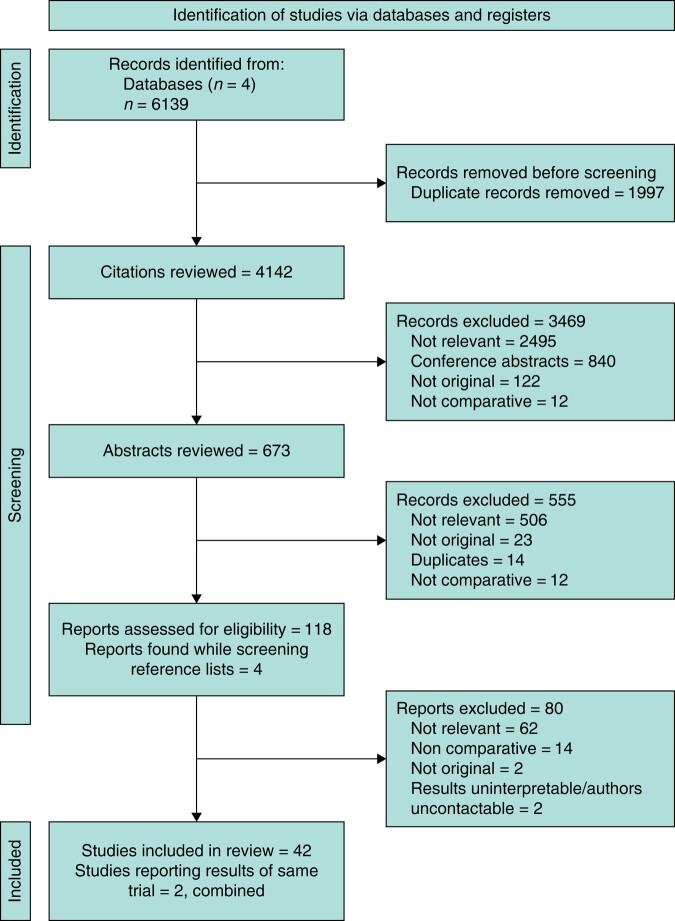
PRISMA flow diagram

In total, 42 papers were identified for inclusion in this review^9,12,28–67^. The 6139 citations retrieved through database searches were screened and a full-text review was performed on 118 papers (*Fig. 1*). The results of an RCT were reported in two separate papers that were combined for the purpose of this review, leaving 41 studies for assessment^62,63^. There was 98.3% agreement between reviewers regarding papers for inclusion (Cohen’s kappa = 0.96).

### Study characteristics

Of the 41 included studies, 38 (92.7%) adopted a pre-/post-intervention cohort design^9,12,28–35,37–60,64–67^, two were randomized trials^36,62,63^ and one was a case–control study^61^. Most (78.9%, *n* = 30) of the pre-/post-intervention studies were quality improvement (QI) projects or audits (*Table S2*)^9,12,28,29,31–35,37–41,43–46,48–51,53,55,56,58,59,65–67^. Only one study received external funding^42^ and most studies (73.2%, *n* = 30) were published within the last 10 years. The majority of studies were carried out in hospitals in the UK (51.2%, *n* = 21)^9,12,28,29,31–35,37–41,44–46,48,53,56,57^ and United States (29.2%, *n* = 12)^30,36,42,50,52,54,58,60–64,66^. More QI projects and audits were carried out in the UK than in the United States (95.2% *versus* 25%, χ^2^ = 14.67, *P* < 0.001).

### Quality assessment

The mean score using the previous QA tool^23^ was 9.2(2.7) out of a maximum of 16 (57.8%). The modified tool assessment of handover research yielded a mean score of 12.3(3.9) out of 23 (53.4%; *Table S3*). The modified tool was better able to distinguish between audits/QI projects and research (new score, 48.7% (15.5) *versus* 66.4% (15); *t* = 3.26, *P* = 0.002; old score, 53.7% (16.1) *versus* 68.7% (14.4), *t* = 2.7, *P* = 0.01). Additionally, the funded study was of better quality, with a QA score of 14.5 compared to the mean of 12.3 (out of 23). QA scores have not improved in the last 10 years (mean modified score, 56.3% (21.2) *versus* 52.1% (15.1); *t* = 0.73, *P* = 0.46; pre- *versus* post-2014).

### Setting and population

Daily end-of-shift handovers were the focus of evaluation in most studies (73.2%, *n* = 30), but weekend handovers were also included (21.9%, *n* = 9). General surgery and orthopaedics had the highest number of studies (*n* = 7, 17.1% each, *Table S2*). Due to the varying nature of outcomes assessed, most studies (*n* = 20; 48.8%) reported patient samples (*n* = 11 946 control *versus n* = 11 563 intervention)^9,29,31–35,41–43,45,46,48,49,51,52,57,59,60,62,63^; however, 14 studies (34.1%) only reported staff samples (*n* = 254 control *versus n* = 238 intervention)^28,30,36–39,44,47,53,56,58,61,64,66^.

### Taxonomy development

#### Handover intervention taxonomy

**Fig. 2:**
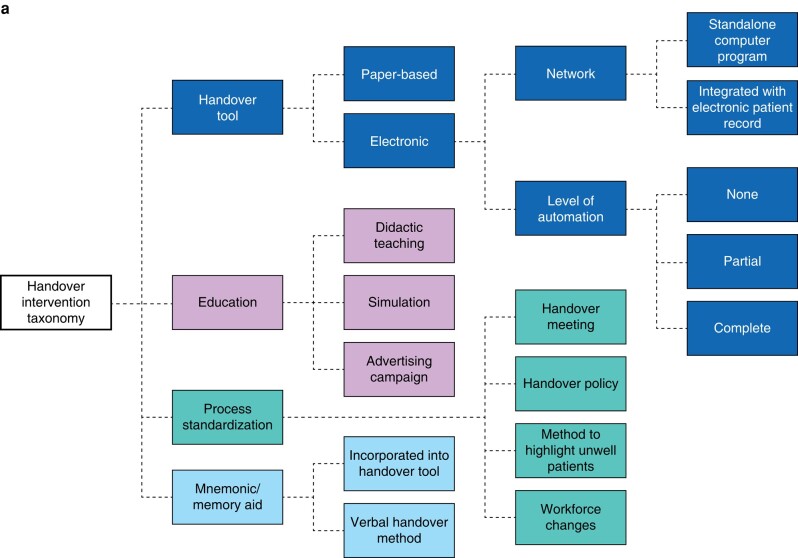

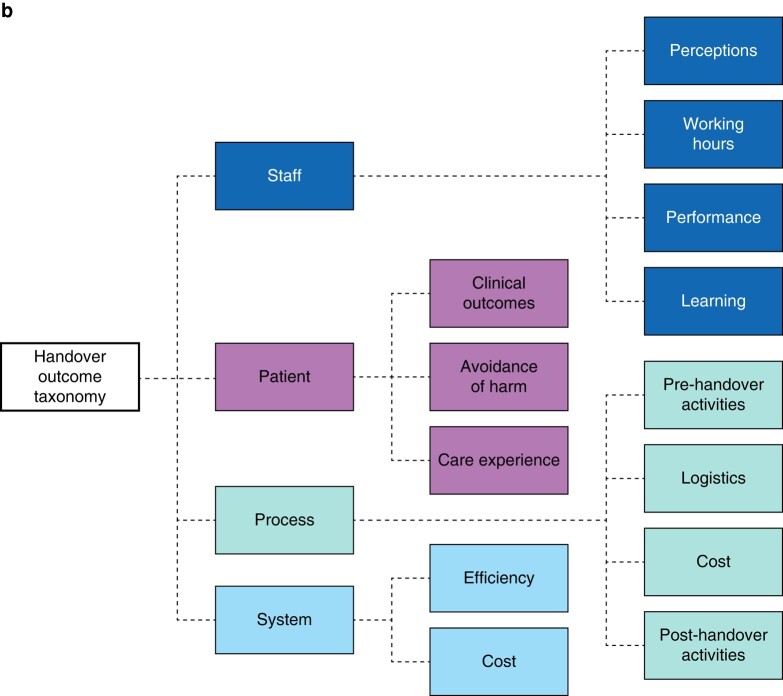
Handover intervention and outcome taxonomy (continued overleaf)

Four categories of handover interventions were developed, including the use of handover tools, process standardization measures, staff education, and the use of a mnemonic or memory aid. Each category includes subcategories; for example, handover tools can be classified as paper *versus* electronic, linked with the electronic patient record *versus* standalone^69^, and by degree of automation^69,70^ (*Fig. 2a*, *Table 2*).

**Table 2:** Intervention definitions and studies utilizing each intervention category

Intervention	Definition	Intervention types	Studies utilizing intervention category	Studies utilizing intervention in isolation
Handover tool	Any paper or electronic instrument Subcategories: Paper or electronic Linked to EPR—yes/no Level of automation—partial/none	Paper or electronic patient list Handover flowchart Handover checklist Mobile phone application	34 (82.9)	11 (26.8)
Process standardization	The implementation of procedures that aim to ensure that handover is carried out consistently within a department or hospital	Handover meeting Handover policy Method of highlighting unwell patients Increased senior supervision	14 (34.1)	1 (2.4)
Staff education	Teaching or training provided to staff either on handover methods, handover policy, or the use of a handover tool	Simulation/role-play Didactic teaching Advertising campaign/posters	25 (60.9)	3 (7.3)
Mnemonic/memory aid	A device that functions either as a memory aid for a handover method, or to structure a handover tool or policy	I-PASS SBAR ABCD	7 (17.1)	0

Values are *n* (%). EPR, electronic patient record; I-PASS, Illness severity, Patient summary, Action list, Situation awareness & contingency planning, Synthesis by receiver; SBAR, Situation, Background, Assessment, Recommendation; ABCD, Airway, Breathing, Circulation, Disability.

#### Handover outcome taxonomy

Outcome categories included patient, staff, process and system outcomes. Process outcomes are differentiated from patient, staff (workforce) or hospital (system) outcomes, recognizing that process improvement does not always correlate with changes in other domains. Each outcome category includes subcategories; for example, patient outcomes can be classified as those relating to care experience, avoidance of harm and clinical outcomes (*Fig. 2b*, *Table 3*).

**Table 3:** Outcome definitions and studies evaluating each outcome category

Outcome	Previous definition^68^	Modified definition	Studies evaluating outcome category	Discrete outcomes in this category (*n*)	Outcomes with significant improvements
Patient	Measures of clinical care processes or outcomes, or derived from patients themselves	Measures that did or could have a direct impact on patient outcomes or the quality of the patient experience	16 (39)	12	7 (58.3)
Process	Not applicable	Measures pertaining to the handover process itself and activities relating to or occurring directly after handover	31 (75.6)	27	14 (51.8)
Staff	Measures derived from staff or pertaining to staff time allocation, efficiency, or other work-related parameters	Measures pertaining directly to staff	15 (36.6)	14	11 (78.5)
System	Measures that characterize a system or technology	Measures that pertain to the institution within which the handover is carried out	3 (7.3)	2	2 (100)

Values are *n* (%).

### Handover interventions (*Table S4*)

Interventions included handover tools (*n* = 34 studies), process standardization (*n* = 14), staff education (*n* = 25) and the use of a mnemonic/memory aid (*n* = 7). Twenty-six (63.4%) studies used a combination of interventions (*Table 2*).

#### Handover tools

Introduction of a tool to support handover constituted the majority of interventions (82.9%, *n* = 34)^9,12,28,29,31,33–35,37–39,41–48,50–53,55–60,62–67^. Tools were classified as either electronic (70.6%, *n* = 24)^28,29,31,34,37,38,42–45,47,48,50,53,55,57–60,62–67^ or paper (29.4%, *n* = 10).^9,12,33,35,39,41,46,51,52,56^. Only one-third (*n* = 8) of electronic tools used any level of automation^28,31,48,58,62,63,65–67^, with a similar proportion (*n* = 8) being linked to the electronic patient record^28,44,50,58,60,62,63,66,67^. The majority (58.3%, *n* = 14) of electronic documents were manually maintained on standalone computer programs (for example, Microsoft Word, Microsoft Excel). Most tools were used in combination with other interventions; however, in more than a quarter of studies (*n* = 11, 26.8%), the introduction of a handover document was the only intervention^31,38,41,45,46,48,50,57,58,64,66^. The introduction of a standardized electronic handover document was most commonly associated with improvements in process and staff outcomes^28,29,31,34,37,38,42,44,50,53,55,57–60,62,64,65,67^. The inclusion of written handover information was the most frequently assessed and improved process-related outcome seen with this intervention^29,31,34,45,57,60,67^. Patient outcomes were not always assessed in these studies; however, improvements in length of stay^42,43,59^, patients missed on ward rounds^62,66^ and infection rates^58^ were reported with the use of a standardized electronic document.

#### Standardization of the handover process

One-third of studies (*n* = 14, 34.1%) utilized some form of handover process standardization^9,12,28,30,32,35,39,43,49,51,52,55,59,65^, all except one^49^ in combination with other interventions. Interventions included a formal face-to-face handover meeting (*n* = 6)^43,49,52,55,59,65^, handover policy (*n* = 5)^12,28,30,35,51^, increased supervision from senior staff (*n* = 1)^32^ and the introduction of a process to highlight high-risk patients (*n* = 3)^9,39,65^. Most studies implementing a new handover meeting demonstrated significant improvements in at least one area, including reduced length of hospital or ICU stay (*n* = 3 and *n* = 1, respectively)^43,52,59^, although increased weekend discharges (*n* = 1)^43^, reduced emergency calls (*n* = 1)^43^, appropriate escalations of care (*n* = 1)^49^ and increased reporting of adverse events (*n* = 1)^65^ were also noted. Introducing a process to identify high-risk patients led to improvements in all studies in which it was tested. One study combined this with a standardized paper handover template and reported a reduction in the average length of stay (LOS) for emergency patients of 1.9 days (*P* = 0.03), increased average weekend discharges (39.1 to 48.9; *P* = 0.003) and putative cost-savings^9^. Another study saw a 147% increase in reporting of adverse events (*P* = 0.007) when a ‘red flag’ system was combined with a new handover meeting^65^. A ‘traffic light system’, supported by staff education and a handover document, reduced ward round duration by 30 min, increased weekend discharges and improved documentation availability and staff confidence (*P* values not reported)^39^. Additionally, increased senior supervision and education increased handover occurrence (*P* < 0.05) and reduced the number of patients with inadequate investigations and treatment (*P* values not reported; calculated by reviewers as *P* < 0.05 and *P* < 0.001, respectively)^32^.

#### Provision of staff education

Twenty-five studies (60.9%) included staff education as all (12%) or part (88%) of the intervention^28–30,32–37,39,40,42–44,47,51,53–56,60–63,65,67^. Simulation was employed in three studies, leading to improved resident handover performance^36^, reduced erroneous order entries (*P* = 0.003)^61^ and a non-significant improvement in staff satisfaction and knowledge of patients^30^.

#### The use of a mnemonic/memory aid to facilitate communication

Mnemonics were used in seven studies (17.1%), including ‘SBAR’ (Situation, Background, Assessment, Recommendation)^28,51,56,61^, ‘I-PASS’ (Illness severity, Patient summary, Action list, Situation awareness & contingency planning, Synthesis by receiver)^60,67^ and ‘ABCD’ (Airway, Breathing, Circulation, Disability)^12^, mostly in combination with staff education and a handover tool (*n* = 6, 85.7%, each). All seven studies using a mnemonic demonstrated improvements in outcomes studied; however, only four studies carried out significance testing^12,60,61,67^. These studies demonstrated improvements in patient (*n* = 1), process (*n* = 3) and staff (*n* = 2) outcomes.

### Outcomes assessed (*Table S4*)

A total of 55 discrete outcomes were assessed in the included studies, categorized as patient (*n* = 12), process (*n* = 27), staff (*n* = 14) and system (*n* = 2) outcomes (*Table 3*). Significant improvements were observed in 51.8%, 78.5%, 58.3% and 100% of the outcomes, respectively. Half of all studies (*n* = 21) evaluated more than one category, with a median of 2 (1–3) outcomes per study.

#### Patient outcomes

The majority of studies evaluating patient outcomes represented level V evidence (*n* = 11, 68.7%)^9,29,32,33,43,48,49,56,58,59,66^. Twelve patient outcomes (*Table 3*) were assessed in 16 studies^9,29,32,33,42,43,48,49,52,56,58–63,66^ with significant improvements in seven outcomes (*n* = 9761 *versus* 9312 patients), including LOS in four studies (*n* = 1635 *versus* 1629 patients)^9,43,52,59^ and ward round review in two studies (*n* = 8018 *versus* 7569 patients; *Table S5*)^62,66^. All four studies demonstrating a significant reduction in LOS utilized a handover tool in combination with a method to standardize the handover process^9,43,52,59^. Increased automation of handover documents led to fewer patients being missed on ward rounds^48,62,66^. Changes were also seen in reasons for transfer^49^, emergency response team calls (*P* < 0.05; *n* = 284 *versus* 310 patients)^43^, inadequate treatment and inadequate investigation (51% *versus* 20%; χ^2^ = 9.6, *P* = 0.0019 and 31.9% *versus* 13.3%; χ^2^ = 4.5; *P* = 0.033, respectively (calculated by reviewers))^32^. Finally, the occurrence of adverse events was assessed in seven studies^29,42,52,58,60,61,63^, with one demonstrating significant improvement^42^. Only one study evaluating adverse events performed a power calculation; however, the power to detect small changes in error rates was relatively low, and this study did not demonstrate any improvement in this area^63^.

#### Process outcomes

In total, 27 process outcomes were evaluated in 31 studies^9,12,28,29,31–35,37–42,44–47,50,51,54,55,57,58,60–62,65–67^, making this the most assessed outcome category (*Table S5*). Handover process outcomes were divided into pre-handover (*n* = 4), logistical (*n* = 10), content-related (*n* = 7) and post-handover (*n* = 6). Content-related outcomes showed the highest levels of improvement (85.7% of outcomes), including completeness of written^9,12,29,31,34,41,45,46,57,60,67^ and verbal^54,67^ handover, number of patients^57^ and tasks^41^ handed over, number of clinical events reported^65^ and transfer of information to nursing staff^44^. Notably, all 11 studies that demonstrated significant improvements in written handover content introduced a handover document as part of the intervention. Significant improvements were also seen in 66.6% of post-handover outcomes, including ward round duration (reduction of 1.5 min per patient; *P* = 0.0006)^62^, time taken to complete documentation (120.3(16.8) s *versus* 37.9(12.4) s; *P* < 0.0001)^50^, erroneous order entries (14.5% *versus* 12.2%; *P* < 0.003)^61^ and availability of information at the bedside (*P* ≤ 0.012)^44^. In contrast, half of all pre-handover outcomes showed improvement, including the time taken to prepare the handover (*P* = 0.012)^44^ and the duration of the pre-round (*P* < 0.0001)^62^. Only 20% of logistical outcomes showed improvement (duration^47,67^ and occurrence^32^ of handover).

#### Staff outcomes

A total of 14 staff outcomes were assessed in 15 studies through staff questionnaires (*n* = 13)^28,30,31,37–39,44,45,47,53,58,64,67^, observation of handovers (*n* = 1)^36^ and measurement of staff overtime (*n* = 1)^12^. The total staff sample sizes for studies with significant findings were 245 (control) *versus* 280 (intervention). No study used a validated questionnaire, although one developed questions through a representative Delphi process^37^ and another was based on national handover guidelines^28^. With various handover interventions, significant improvements were seen in handover performance^36^, staff overtime^12^, staff perception of handover quality^58,60,64^, staff satisfaction^67^, perceived handover safety^58^, perceived process efficiency^58^, perceived service coordination impact^31^, perceived ward round efficiency^44^, perceived information governance^44^, staff knowledge of patients^47^ and clarity of transfer of responsibility (*Table S5*).

#### System outcomes

Weekend discharges were evaluated in three studies, two of which demonstrated significant improvement^9,43^ and one that reported improvement without *P* values (5% *versus* 20%)^39^. One study extrapolated cost savings of £740 000 and £3.82 million arising from a reduced LOS after the introduction of a handover intervention^9^.

## Discussion

Of the 6139 screened citations, 41 studies of 23 509 patients were identified, which evaluated the impact of four categories of handover interventions on 55 discrete outcomes. Interventions were mostly tool-based (82.9%), with mixed interventions being common (63.4%). Outcomes were widely heterogeneous and rigorously evaluated in a minority of studies, with only two RCTs^36,62,63^ and one case–control study^61^. Even by the standards of earlier, less-rigorous QA tools, more than half of the studies met fewer than 65% of quality metrics. A novel taxonomy for the interventions and outcomes used in handover research was developed and the existing handover research QA tool was revised to increase its rigor. As expected, a meta-analysis was not possible because of the clinical and methodological heterogeneity between studies^22^.

Multiple varying taxonomies of handover interventions and outcomes have been described previously^18,69,70,71^. These variations reflect the heterogeneity of the literature and lack of methodological guidance for handover research. Novel taxonomies for handover research interventions and outcomes were developed, which will help reduce heterogeneity in future research.

A modified methodological tool for assessing the quality of handover research is also reported in this paper. The original tool omitted items relating to internal validity, quality of reporting and power calculations^23^ and the modified tool better differentiated high- from low-quality studies. Quality scores have not improved since the last systematic review on this topic 10 years ago^18^, despite a large increase in studies. However, the standard of surgical handover research is comparable to healthcare handover research generally, which has a median score of 9–10^23,70,72^. The use of the modified tool will support better-quality future research in all areas of handover research beyond the discipline of surgery alone.

Handover tools, specifically documents, were the most common interventions, and electronic documents were often associated with process outcomes, including improvements in written handover content and some patient outcomes. One-quarter of all papers used a handover document as the only intervention, the majority of which (63.6%) were published within the last 10 years. A handover involves information transfer about a patient from one doctor to another^4^, but more importantly requires two-way communication^73^, which cannot be guaranteed with a simple document. Automation has not been adequately tested here, with a minority of electronic tools utilizing it^28,31,48,58,62,63,65–67^. Manual updating of a tool hampered handover in multiple studies^70^ and can contribute significantly to written errors^74^. Facilitating automation in future studies would reduce wasted effort and improve staff experience. While staff education was utilized in most studies^28–30,32–37,39,40,42–44,47,51,53–56,60–63,65,67^, interventions were poorly described, with only one study reporting methodology to a replicable degree^61^. Surgical handover curricula would benefit from increased research in educational interventions.

Regarding process standardization, both the introduction of a handover meeting and a method to highlight unwell patients demonstrated improvements. The I-PASS handover bundle specifically requires that illness severity is highlighted at the beginning of each patient presentation and significantly reduces preventable adverse events^75^. Drawing the listener’s attention to the sickest patients on the list should be a vital component of any future interventions.

Surgical handover interventions led to significant improvements in 58.3% of patient outcomes. Cohen and Hilligoss (2010) describe handover practices as deeply embedded in local culture and remark that staff are unlikely to change their behaviour unless they see concrete improvements in patient outcomes^4^. Focusing on patient outcomes in future studies is worthwhile; however, measurement is laborious, time-consuming, and often requires funding. Starmer *et al*. required two research nurses for 5 days/week to identify a reduction in preventable adverse events^75^. In the current review, seven studies assessed adverse events^29,42,52,58,60,61,63^ but only one demonstrated improvement^58^. Only one study performed a power calculation^63^ and data collection methods were variable. Despite the relatively high number of studies evaluating this outcome, a reduction in adverse events with an improved study design cannot be reliably predicted. In addition to adverse events, LOS and ward round reviews appear to be key outcomes for assessment in handover research.

The rate of funded handover research (2.4%) is much lower in surgery than in healthcare overall (15–28.6%)^69,72^. Funded studies receive higher QA scores^72,76^, as reflected in this review^42^. Poorly conducted handovers are expensive in terms of opportunity costs associated with inefficient processes, wasted staff time and adverse events. Surprisingly, no studies included any form of cost/benefit analysis, workforce cost implications, or evaluation of the impact of handover related to risk management or medicolegal claims. The monetary cost of communication failures^3^ and potential cost savings of handover interventions^9^ speak to the potential return on investment in funding higher-quality handover research.

The main limitation of this review is the poor quality of available data. Most studies are level V evidence; even when assessed using tools that accept the limitations inherent in handover research, quality scores remain low. Only one study performed a power calculation^62,63^, 43.9% did not perform significance testing for at least one outcome^12,28,30,32,33,35,37–40,42,45,48,51,53,55,56,58^ and 21.9% did not report at least one sample size^9,30,33,45,47,55,58,65,67^. The existing QA tool for handover research was updated to incorporate key tenets in assessing research quality in order to more accurately distinguish between high- and low-quality studies, and to raise standards for future research.

The universal lack of accepted outcome measures for handover research has led to a wide variety of interventions and outcomes being used, including 11 different combinations of interventions and 9 different combinations of outcomes. As such, it was not possible to directly compare many of the studies, and the subject was not suitable for meta-analysis. Prioritization of interventions and outcomes for handover research, through the development of a core outcome set, would minimize variation in the future. In addition, the subject area would benefit from specific reporting guidelines.

Despite the implications of daily surgical handover in terms of patient safety, staff workflow and hospital expenditure, a body of supportive interventional research has yet to be established. It is important to mitigate the risks associated with handover through process improvements. However, unplanned disruption of existing workflow patterns may increase harm; therefore, it is necessary to demonstrate that new approaches both improve patient safety and deliver value to the health system. At present, effective interventions appear to include implementing a formal face-to-face handover meeting, an automated electronic handover document listing patient details, a method to highlight critically unwell patients, ensuring appropriate senior supervision, staff education and the use of a mnemonic or memory aid to structure patient presentations. Future studies should prioritize these interventions and their effect on patient outcomes, particularly adverse events, ward round reviews and length of stay. The novel taxonomies described here also provide a new language with which to describe handover research and create uniformity in future research studies.

## Supplementary Material

zrae011_Supplementary_Data

## Data Availability

Additional data have not been published in a public repository. The authors agree to make the data, analytic methods and study materials available to other researchers. These can be obtained by contacting the corresponding author using the details provided.
