# Supplementary material for: Daily handover in surgery: systematic review and a novel taxonomy of interventions and outcomes
Source: BJS Open. 2024 Mar 1;8(2):zrae011. doi: 10.1093/bjsopen/zrae011 (PMC10905088; doi:10.1093/bjsopen/zrae011)
Supplement: zrae011_Supplementary_Data [file zrae011_supplementary_data.zip › Appendix_S1._Search_strategy.pdf]

Ovid®

Wolters Kluwer

My Account | Support & Training | Help | Feedback | Logged in as [redacted] | Logoff

Search | Journals | Books | Multimedia | **My Workspace** | Visible Body | What's New

My Projects | My Searches & Alerts | My eTocs | Send us your feedback

Edit Search

Search Name: A systematic review of interventions to improve daily handover in surgery

Comment:

Save Cancel

Set	Search Statement	Annotations	Insert	Edit	Delete
1.	specialties, surgical/ or colorectal surgery/ or general surgery/ or gynecology/ or neurosurgery/ or obstetrics/ or ophthalmology/ or orthognathic surgery/ or orthopedics/ or otolaryngology/ or surgery, plastic/ or surgical oncology/ or thoracic surgery/ or traumatology/ or urology/				
2.	surgery.mp.				
3.	surgeon.mp.				
4.	surgical.mp.				
5.	1 or 2 or 3 or 4				
6.	information transfer.mp.				
7.	(information adj1 transfer).mp. [mp=title, book title, abstract, original title, name of substance word, subject heading word, floating sub-heading word, keyword heading word, organism supplementary concept word, protocol supplementary concept word, rare disease supplementary concept word, unique identifier, synonyms]				
8.	information flow.mp.				
9.	(information adj1 flow).mp. [mp=title, book title, abstract, original title, name of substance word, subject heading word, floating sub-heading word, keyword heading word, organism supplementary concept word, protocol supplementary concept word, rare disease supplementary concept word, unique identifier, synonyms]				
10.	6 or 7				
11.	8 or 9				
12.	hand over.mp.				
13.	handover.mp.				
14.	(hand adj1 over).mp. [mp=title, book title, abstract, original title, name of substance word, subject heading word, floating sub-heading word, keyword heading word, organism supplementary concept word, protocol supplementary concept word, rare disease supplementary concept word, unique identifier, synonyms]				
15.	12 or 13 or 14				
16.	Patient Handoff/				
17.	handoff.mp.				
18.	hand off.mp.				
19.	(hand adj1 off).mp. [mp=title, book title, abstract, original title, name of substance word, subject heading word, floating sub-heading word, keyword heading word, organism supplementary concept word, protocol supplementary concept word, rare disease supplementary concept word, unique identifier, synonyms]				
20.	17 or 18 or 19				
21.	signoff.mp.				
22.	sign off.mp.				
23.	(sign adj1 off).mp. [mp=title, book title, abstract, original title, name of substance word, subject heading word, floating sub-heading word, keyword heading word, organism supplementary concept word, protocol supplementary concept word, rare disease supplementary concept word, unique identifier, synonyms]				
24.	signout.mp.				
25.	sign out.mp.				
26.	(sign adj1 out).mp. [mp=title, book title, abstract, original title, name of substance word, subject heading word, floating sub-heading word, keyword heading word, organism supplementary				

concept word, protocol supplementary concept word, rare disease supplementary concept word, unique identifier, synonyms]

27. 21 or 22 or 23
28. 24 or 25 or 26
29. "Continuity of Patient Care"/
30. ISBAR.mp.
31. ISBAR communication tool.mp.
32. (ISBAR adj1 communication adj1 tool).mp. [mp=title, book title, abstract, original title, name of substance word, subject heading word, floating sub-heading word, keyword heading word, organism supplementary concept word, protocol supplementary concept word, rare disease supplementary concept word, unique identifier, synonyms]
33. (introduction adj1 situation adj1 background adj1 assessment adj1 recommendation).mp. [mp=title, book title, abstract, original title, name of substance word, subject heading word, floating sub-heading word, keyword heading word, organism supplementary concept word, protocol supplementary concept word, rare disease supplementary concept word, unique identifier, synonyms]
34. SBAR.mp.
35. 30 or 31 or 32 or 33 or 34
36. (clinical adj1 handover adj1 assessment adj1 tool).mp. [mp=title, book title, abstract, original title, name of substance word, subject heading word, floating sub-heading word, keyword heading word, organism supplementary concept word, protocol supplementary concept word, rare disease supplementary concept word, unique identifier, synonyms]
37. I-PASS.mp.
38. communication tool.mp.
39. (communication adj1 tool).mp. [mp=title, book title, abstract, original title, name of substance word, subject heading word, floating sub-heading word, keyword heading word, organism supplementary concept word, protocol supplementary concept word, rare disease supplementary concept word, unique identifier, synonyms]
40. communication aid.mp.
41. (communication adj1 aid).mp. [mp=title, book title, abstract, original title, name of substance word, subject heading word, floating sub-heading word, keyword heading word, organism supplementary concept word, protocol supplementary concept word, rare disease supplementary concept word, unique identifier, synonyms]
42. 40 or 41
43. 38 or 39
44. briefing.mp.
45. checklist.mp. or Checklist/
46. 10 or 11 or 15 or 16 or 20 or 27 or 28 or 29 or 35 or 36 or 37 or 42 or 43 or 44 or 45
47. 5 and 46
48. 10 or 11 or 15 or 16 or 20 or 27 or 28 or 29 or 35 or 36 or 37 or 42 or 43 or 44
49. 5 and 48

Save Cancel

English Français Italiano Deutsch 日本語 繁體中文 Español 简体中文 한국어

About Us Contact Us Privacy Policy Terms of Use

© 2022 Ovid Technologies, Inc. All rights reserved. OvidUI_04.20.01.001, SourceID bab09fc86e1540d01875b74c93f5fdf6f33181f0
