# Supplementary material for: Daily handover in surgery: systematic review and a novel taxonomy of interventions and outcomes
Source: BJS Open. 2024 Mar 1;8(2):zrae011. doi: 10.1093/bjsopen/zrae011 (PMC10905088; doi:10.1093/bjsopen/zrae011)
Supplement: zrae011_Supplementary_Data [file zrae011_supplementary_data.zip › Supplementary_Material.docx]

**TITLE PAGE**

**A systematic review of daily handover in surgery: a novel taxonomy of interventions and outcomes**

Jessica M. Ryan, MRCSI^1, 2, 3^, Fiachra McHugh, MRCSI^4^, Anastasija Simiceva MSc^1^, Walter Eppich, PHD^1^, Dara O. Kavanagh, FRCS^5, 6^, Deborah A. McNamara, FRCS^7, 8, 9^

**Author institutions**

^1^ RCSI SIM Centre for Simulation Education and Research, 123 St. Stephen’s Green, Co. Dublin

^2^ RCSI StAR MD programme, St. Stephen’s Green, Co. Dublin

^3^ The Bon Secours Hospital, Glasnevin Hill, Glasnevin, Co. Dublin

^4^ Department of Surgery, Mayo University Hospital, Castlebar, Co. Mayo

^5^ RCSI Department of Surgical Affairs, 121 St. Stephen’s Green, Co. Dublin

^6^ Department of Surgery, Tallaght University Hospital, Tallaght, Co. Dublin

^7^ Office of the President, RCSI, 123 St. Stephen’s Green, Co. Dublin

^8^ National Clinical Programme in Surgery, RCSI, 2 Proud’s Lane, Co. Dublin

^9^ Department of Surgery, Beaumont Hospital, Beaumont, Co. Dublin

**CORRESPONDING AUTHOR**

Name: Jessica M Ryan

Address: RCSI SIM Centre for Simulation Education and Research, 123 St. Stephen’s Green, Co. Dublin, Ireland

Email: [jessicaryan@rcsi.com](mailto:jessicaryan@rcsi.com)

ORCID: <https://orcid.org/0000-0001-6161-9630>

Twitter: @jessmryan

Telephone number: +35314022100

Fax number: None available

**Supplementary Materials – Index**

| **Supplementary Appendixes** | |  |
| --- | --- | --- |
| Appendix S1. Search strategy | *Supplied as separate PDF* | |
|  | |  |
| **Supplementary Figures and Tables** | |  |
| Table S1. Modified Quality Scoring System for Evaluation of Handover Research Studies  Table S2. Study characteristics  Table S3. Quality assessment scores  Table S4. Study interventions, outcomes, and results  Table S5a, b, c. Patient, process, and staff outcomes | | *Pg. 4*  *pg. 5-8*  *pg. 9-10*  *pg. 11-17*  *pg. 19-22* |
|  | |  |
| **References** | | *Pg. 23-25* |
|  | |  |

**Supplementary Figures and Tables**

**Table S1.** Modified Quality Scoring System for Evaluation of Handover Research Studies

| **Study type** | **Points** |  |  |
| --- | --- | --- | --- |
| Single group cross sectional, or single group post-test only or qualitative study | 1 |  |  |
| Single group pre and post-test, or cohort | 1.5 |  |  |
| Nonrandomized trial (includes control or comparison group) | 2 |  |  |
| Randomized controlled trial | 3 |  |  |
| **Total sample size** |  |  |  |
| Unclear | 0 |  |  |
| ≤10 | 0.5 |  |  |
| 11 to 50 | 1 |  |  |
| 51 to 100 | 1.5 |  |  |
| 101 to 150 | 2 |  |  |
| 151 to 200 | 2.5 |  |  |
| 201 or more | 3 |  |  |
| **Reporting** | **Yes** | **No** | **Unable to determine** |
| Is the hypothesis/aim/objective/purpose of the study clearly described? | 1 | 0 |  |
| Are the participants clearly described? | 1 | 0 |  |
| Are the main outcomes to be measured clearly describe in the Introduction or Methods section? | 1 | 0 |  |
| Are the methods described with enough details to replicate the study? **Note - if a handover tool was implemented, a copy should be provided** | 1 | 0 |  |
| Are the main **findings** of the study clearly described in the Results? | 1 | 0 |  |
| **Have actual probability values been reported (e.g., 0.035 rather than <0.05) for the main outcomes except where the probability value is less than 0.001?** | 1 | 0 |  |
| **Internal validity** |  |  |  |
| Did they use a previously validated or published instrument, questionnaire, interview script? **If not applicable, were published guidelines used to audit practice? (content of assessment proforma must be taken directly from guidelines)** | 1 | 0 |  |
| Did they conduct any validity assessment? | 1 | 0 |  |
| Did they use any method designed to enhance the quality measurement? | 1 | 0 |  |
| Did they report obtaining Institutional Review Board (IRB) approval? | 1 | 0 |  |
| Did the reported conclusions follow from the reported results? | 1 | 0 |  |
| **Was an attempt made to blind those measuring the main outcomes of the intervention?** | 1 | 0 | 0 |
| **Were the statistical tests used to assess the main outcomes appropriate?** | 1 | 0 | 0 |
| **Was staff compliance with the intervention/s reliable?** | 1 | 0 | 0 |
| **Were study subjects randomised to intervention groups?** | 1 | 0 | 0 |
| **Was there adequate adjustment for confounding in the analyses from which the main findings were drawn?** | 1 | 0 | 0 |
| **Power** |  |  |  |
| **Was a power calculation performed?** | 1 | 0 |  |

*Adapted from Riesenberg et al., 2009^1^ and Downs and Black, 1998^2^ with changes underlined, bolded, and in red

**Table S2.** Study characteristics

| First author | Year | Country | Study design | Hospital type | Beds | Specialty | Definition of handover | Funding |
| --- | --- | --- | --- | --- | --- | --- | --- | --- |
| Advani | 2015 | UK | Multi-site QI project  BMA 2004 guidelines | Three sites covering the trust:  One DGH & two  community hospitals | 513 | Surgery (multiple specialties) | Daily end-of-shift handover | No |
| Ahmed | 2013 | UK | Prospective audit  RCSE 2007 guidelines | DGH | 556 | Acute surgery | Handover of all acute surgical admissions | No |
| Antonoff | 2012 | USA | Pre-/post intervention cohort | University teaching hospital & Level II Trauma centre | 1700 | Surgery (multiple specialties) | Transitions of care for surgical inpatients | No |
| Bakti | 2017 | UK | Prospective audit  BMA 2004 guidelines | DGH | 478 | Orthopaedic surgery | Daily end-of-shift handover (morning) of new admissions | No |
| Bass | 2015 | UK | QI project  RCSE 2007 guidelines | DGH | 749 | Urology | Daily end-of shift handover (morning) from the general surgical to the urological team | No |
| Bethune | 2014 | UK | QI project  Guidelines nr | University teaching hospital | 843 | Surgery and medicine | Handover of inpatients to the weekend on-call team | No |
| Blower | 2014 | UK | Audit  RCSE 2007 guidelines | DGH | 625 | General surgery | Daily end-of-shift handover (post-take) | No |
| Bradley | 2014 | UK | QI project  RCSE 2007 guidelines | DGH | 267 | Emergency surgery | Handover of patients from the Combined Assessment Unit to the surgical ward (between 0800 and 2200 hours) | No |
| Britt | 2015 | USA | RCT | University Teaching Hospital | 555 | Surgery & paediatrics | Handover of patient information to a simulated resident | No |
| Chopra | 2016 | UK | Prospective audit  Guidelines nr | DGH | 500 | General surgery | Daily end-of-shift handover (morning and evening) | No |
| Culwick | 2014 | UK | QI project  Guidelines nr | DGH | 400 | General surgery, Urology | Handover to the weekend on-call team | No |
| Dean | 2017 | UK | QI project  RCSE 2007 guidelines | DGH | 518 | General surgery | Handover to the weekend on-call team | No |
| Din | 2012 | UK | QI Project  RCSE 2007 guidelines | DGH | 400 | General & Vascular surgery | Handover of general surgical patients to the weekend on-call team | No |
| Ellul | 2011 | UK | Prospective audit  RCSE 2007 guidelines | DGH | 500 | ENT | Daily end-of-shift handover (morning and afternoon) | No |
| Ferran | 2008 | UK | Prospective audit  RCSE 2007 guidelines | University teaching hospital | 1080 | Orthopaedic surgery | End-of-shift handover of new admissions | No |
| Gagnier | 2016 | USA | Pre-/post intervention cohort | University teaching hospital | 550 | Orthopaedic surgery | All handovers of adult orthopaedic patients | Yes |
| Gibbons | 2015 | IRE | Prospective audit  Guidelines nr | University teaching hospital | 562 | General surgery, Vascular | Handover to the weekend on-call team | No |
| Jacob | 2021 | UK | QI project  Guidelines nr | DGH | 419 | Surgery (ns) | Daily end-of-shift handover (0800 & 2000 hours) | No |
| Jardine | 2014 | UK | QI project  Guidelines nr | University teaching hospital | 500 | Medicine and surgery | Weekend handover of inpatients | No |
| Jones | 2015 | UK | Prospective audit  RCSE 2007 | DGH | 463 | General Surgery | Daily end-of-shift handover of new admissions (twice per day) | No |
| Khanna | 2015 | India | Pre-/post intervention cohort | Teaching hospital & Quaternary referral centre | 300 | Orthopaedic Surgery | Daily end-of-shift handover (morning) | No |
| Khoury | 2018 | UK | Audit:  Guidelines nr | DGH | 740 | Orthopaedic Surgery | Handover of postoperative patients over the weekend | No |
| Krishna | 2018 | NZ | QI project  Guidelines nr | Centre for elective surgery | 78 | Surgery (multiple specialties) | Daily end-of-shift handover (1500 & 2200 hours) | No |
| Krushelnytskyy | 2022 | USA | QI project  Guidelines nr | University teaching hospital | 943 | Neurosurgery | Daily end-of-shift handover (morning) | No |
| Law | 2022 | AUS | QI project  Local (hospital) guidelines | General hospital,  Secondary care centre | 500 | Medical and Surgical teams | Daily end-of-shift (out of hours) handover (0800 & 2100 hours) | No |
| Lee | 2014 | USA | Pre-/post intervention cohort | Teaching, regional hospital & Level II Trauma centre | 631 | Trauma surgery | Daily end-of-shift handover (morning) | No |
| Maroo | 2017 | UK | QI project   - RCSE, RCPE | DGH | 391 | Orthopaedic surgery | Handover to the weekend on-call team | No |
| Ottinger | 2017 | USA | Pre/post-intervention cohort | University teaching hospital & Level I Trauma centre | 719 | Trauma surgery | End-of-shift handover (morning) | No |
| Piscioneri | 2011 | AUS | Pre/post-intervention cohort | University teaching hospital | 672 | Surgery (multiple specialties) | Weekday end-of-shift handover (morning) | No |
| Ramsay | 2018 | UK | QI project   - Guidelines nr | University teaching hospital | 862 | Emergency surgery | Handover from the Acute Surgical Receiving Unit to the General Ward | No |
| Raptis | 2009 | UK | Pre-/post intervention cohort | University teaching hospital | 600 | Nursing, medicine, and surgery | Handover from day to night staff | No |
| Raval | 2015 | USA | QI project   - nr | University teaching hospital & Quaternary referral centre for paediatric surgery | 450 | Paediatric surgery | nr | No |
| Ryan | 2010 | IRE | Prospective audit   - RCSE 2007 | University teaching hospital | 562 | General Surgery | Daily end-of-shift handover (morning) | No |
| Sadri | 2013 | UK | Prospective audit   - RCSE 2007 | University teaching hospital | 430 | Plastic surgery | Daily end-of-shift handover (evening) |  |
| Stenquist | 2022 | USA | Multi-site pre-/post intervention cohort | Two University teaching hospitals & Level I trauma centres | 999  793 | Orthopaedic surgery | Daily end-of-shift handover | No |
| Telem | 2011 | USA | Non-randomised case control study | University teaching hospital | 1141 | General surgery | Simulated handovers | No |
| Van Eaton | 2005 & 2010 | USA | Multi-site prospective, randomised, crossover study | Level I trauma centre  University Teaching Hospital | 368  450 | Surgery &  Internal Medicine | Daily end-of-shift handover (morning) | No |
| Wayne | 2008 | USA | Pre-/Post-intervention cohort | University teaching hospital | 903 | Surgery (multiple specialties) | Daily end-of-shift handover | No |
| White-Gibson | 2017 | IRE | Prospective audit  NCEC 2015 & RCSE 2007 guidelines | University teaching hospital | 562 | General surgery | Handover to the weekend on-call team | No |
| Wohlauer | 2011 | USA | QI Project  Guidelines nr | University teaching hospital | 678 | Medicine and surgery | Daily end-of-shift handovers | No |
| Wolinska | 2021 | CAN | QI Project  I-PASS handover method | University teaching hospital. Paediatric tertiary referral and level I trauma centre | 453 | Paediatric surgery | Daily end-of-shift handovers | No |

*UK, United Kingdom; QI, Quality Improvement; DGH, District General Hospital; USA, United States of America; nr, Not Reported; CAN, Canada; IRE, Ireland; AUS, Australia; NCEC, National Clinical Effectiveness Committee; RCSE, Royal College of Surgeons in England; I-PASS, Illness severity, patient summary, action list, situation awareness and contingency planning, synthesis by receiver; RCPE, Royal College of Physicians of England; BMA, British Medical Association

**Table S3.** Quality assessment scores

| Author | Year | Study type | Sample size | Hypothesis/ aim/ objective/ purpose | Participants | Out-comes | Methods | Findings | Validated/ published instrument | Validity assess-ment | Enhance quality of measure-ment | Ethics | Con-clusions | Probability values | Blinding | Stats | Staff compliance | Random-isation | Con-founders | Power calculation | Old score | New score |
| --- | --- | --- | --- | --- | --- | --- | --- | --- | --- | --- | --- | --- | --- | --- | --- | --- | --- | --- | --- | --- | --- | --- |
| Ferran | 2008 | 1.5 | 2 | 1 | 0 | 0 | 0 | 1 | 1 | 0 | 0 | 0 | 1 | 0 | 0 | 1 | 1 | 0 | 0 | 0 | 7.5 | 10.5 |
| Ryan | 2010 | 1.5 | 1.5 | 1 | 0 | 1 | 1 | 1 | 0 | 0 | 1 | 0 | 1 | 1 | 0 | 1 | 1 | 0 | 1 | 0 | 9 | 14 |
| Piscioneri | 2011 | 0 | 0 | 0 | 0 | 0 | 1 | 1 | 0 | 0 | 0 | 0 | 0 | 0 | 0 | 0 | 1 | 0 | 0 | 0 | 2 | 3 |
| Ellul | 2011 | 1.5 | 1 | 1 | 0 | 1 | 0 | 1 | 1 | 0 | 1 | 0 | 1 | 0 | 1 | 0 | 1 | 0 | 0 | 0 | 8.5 | 11.5 |
| Wohlauer | 2011 | 1.5 | 1.5 | 1 | 1 | 1 | 1 | 1 | 1 | 0 | 0 | 0 | 1 | 1 | 0 | 1 | 1 | 0 | 0 | 0 | 10 | 14 |
| Din | 2012 | 1.5 | 1 | 1 | 1 | 1 | 1 | 0 | 0 | 0 | 0 | 0 | 1 | 0 | 0 | 0 | 0 | 0 | 0 | 0 | 7.5 | 8.5 |
| Ahmed | 2012 | 1.5 | 3 | 1 | 1 | 1 | 0 | 1 | 1 | 0 | 1 | 1 | 1 | 1 | 1 | 1 | 1 | 0 | 1 | 0 | 12.5 | 18.5 |
| Sadri | 2013 | 1.5 | 1 | 1 | 0 | 1 | 1 | 1 | 1 | 0 | 0 | 0 | 0 | 0 | 0 | 1 | 1 | 0 | 0 | 0 | 7.5 | 9.5 |
| Bethune | 2014 | 1.5 | 0 | 1 | 0 | 1 | 0 | 0 | 0 | 0 | 0 | 0 | 0 | 0 | 0 | 0 | 1 | 0 | 0 | 0 | 3.5 | 4.5 |
| Jardine | 2014 | 1.5 | 0 | 0 | 0 | 1 | 0 | 1 | 0 | 0 | 0 | 0 | 1 | 0 | 0 | 0 | 1 | 0 | 0 | 0 | 4.5 | 5.5 |
| Culwick | 2014 | 1.5 | 1 | 0 | 1 | 0 | 0 | 1 | 0 | 0 | 1 | 0 | 0 | 0 | 0 | 0 | 1 | 0 | 0 | 0 | 5.5 | 7.5 |
| Bradley | 2014 | 1.5 | 2 | 1 | 1 | 1 | 0 | 1 | 1 | 0 | 1 | 0 | 1 | 0 | 0 | 0 | 1 | 0 | 0 | 0 | 10.5 | 12.5 |
| Blower | 2014 | 1.5 | 3 | 1 | 1 | 1 | 0 | 1 | 1 | 0 | 1 | 0 | 1 | 1 | 1 | 1 | 0 | 0 | 0 | 0 | 11.5 | 15.5 |
| Raval | 2015 | 1.5 | 1 | 1 | 0 | 1 | 1 | 1 | 0 | 0 | 0 | 0 | 1 | 0 | 0 | 1 | 0 | 0 | 0 | 0 | 7.5 | 8.5 |
| Gibbons | 2015 | 1.5 | 3 | 1 | 0 | 1 | 0 | 1 | 0 | 0 | 0 | 0 | 1 | 0 | 0 | 1 | 0 | 0 | 0 | 0 | 8.5 | 10.5 |
| Bass | 2015 | 1.5 | 1.5 | 1 | 1 | 1 | 1 | 1 | 0 | 0 | 0 | 1 | 1 | 0 | 0 | 0 | 0 | 0 | 0 | 0 | 10 | 11 |
| Advani | 2015 | 1.5 | 1 | 1 | 1 | 1 | 0 | 1 | 1 | 0 | 1 | 1 | 1 | 0 | 0 | 0 | 1 | 0 | 1 | 0 | 10.5 | 13.5 |
| Jones | 2015 | 1.5 | 2 | 1 | 0 | 1 | 1 | 1 | 1 | 0 | 0 | 0 | 1 | 1 | 0 | 1 | 1 | 0 | 0 | 0 | 9.5 | 13.5 |
| Chopra | 2016 | 1.5 | 1 | 1 | 1 | 1 | 1 | 1 | 1 | 0 | 1 | 0 | 1 | 0 | 0 | 0 | 1 | 0 | 0 | 0 | 10.5 | 12.5 |
| Maroo | 2017 | 1.5 | 0.5 | 1 | 0 | 1 | 0 | 1 | 0 | 0 | 0 | 0 | 0 | 0 | 0 | 0 | 1 | 0 | 0 | 0 | 5 | 7 |
| White-Gibson | 2017 | 1.5 | 1 | 1 | 0 | 1 | 0 | 1 | 0 | 0 | 1 | 0 | 1 | 1 | 1 | 1 | 1 | 0 | 0 | 0 | 7.5 | 11.5 |
| Dean | 2017 | 1.5 | 3 | 1 | 0 | 1 | 1 | 1 | 1 | 0 | 0 | 1 | 1 | 1 | 0 | 1 | 1 | 0 | 0 | 0 | 11.5 | 14.5 |
| Bakti | 2017 | 1.5 | 3 | 1 | 1 | 1 | 1 | 1 | 1 | 0 | 1 | 0 | 1 | 1 | 0 | 1 | 1 | 0 | 0 | 0 | 12.5 | 16.5 |
| Khoury | 2018 | 1.5 | 1.5 | 1 | 0 | 1 | 1 | 1 | 0 | 0 | 0 | 0 | 1 | 0 | 0 | 0 | 0 | 0 | 0 | 0 | 8 | 9 |
| Author | Year | Study type | Sample size | Hypothesis/ aim/ objective/ purpose | Participants | Out-comes | Methods | Findings | Validated/ published instrument | Validity assess-ment | Enhance quality of measure-ment | Ethics | Con-clusions | Probability values | Blinding | Stats | Staff compliance | Random-isation | Con-founders | Power calculation | Old score | New score |
| Ramsay | 2018 | 1.5 | 1 | 1 | 1 | 1 | 1 | 1 | 1 | 0 | 0 | 0 | 1 | 0 | 0 | 0 | 0 | 0 | 0 | 0 | 9.5 | 10.5 |
| Krishna | 2018 | 1.5 | 2 | 1 | 0 | 1 | 0 | 1 | 0 | 0 | 0 | 1 | 1 | 1 | 0 | 1 | 0 | 0 | 1 | 0 | 8.5 | 11.5 |
| Jacob | 2021 | 1.5 | 1.5 | 1 | 0 | 1 | 1 | 1 | 0 | 0 | 0 | 0 | 1 | 1 | 0 | 0 | 1 | 0 | 0 | 0 | 8 | 11 |
| Wolinska | 2021 | 1.5 | 1 | 1 | 1 | 1 | 1 | 1 | 1 | 0 | 1 | 1 | 1 | 0 | 0 | 1 | 1 | 0 | 0 | 0 | 11.5 | 13.5 |
| Law | 2022 | 1.5 | 1 | 1 | 0 | 1 | 1 | 1 | 1 | 0 | 0 | 0 | 1 | 0 | 0 | 0 | 1 | 0 | 0 | 0 | 8.5 | 10.5 |
| Krushelnytskyy | 2022 | 1.5 | 2.5 | 1 | 1 | 1 | 1 | 1 | 0 | 0 | 0 | 1 | 1 | 1 | 0 | 1 | 1 | 0 | 1 | 0 | 11 | 16 |
| Wayne | 2008 | 1.5 | 2.5 | 1 | 0 | 1 | 1 | 1 | 0 | 1 | 1 | 0 | 1 | 1 | 0 | 1 | 0 | 0 | 1 | 0 | 11 | 15 |
| Raptis | 2009 | 1.5 | 3 | 1 | 0 | 1 | 0 | 1 | 0 | 0 | 0 | 0 | 0 | 1 | 1 | 1 | 1 | 0 | 0 | 0 | 7.5 | 12.5 |
| Antonoff | 2013 | 1.5 | 1 | 1 | 1 | 1 | 0 | 1 | 0 | 0 | 1 | 1 | 0 | 0 | 0 | 1 | 1 | 0 | 1 | 0 | 8.5 | 11.5 |
| Lee | 2014 | 1.5 | 3 | 1 | 1 | 1 | 1 | 1 | 0 | 0 | 0 | 0 | 1 | 0 | 0 | 1 | 0 | 0 | 0 | 0 | 10.5 | 12.5 |
| Khanna | 2015 | 1.5 | 0.5 | 1 | 0 | 1 | 1 | 1 | 0 | 0 | 0 | 1 | 1 | 1 | 1 | 0 | 1 | 0 | 0 | 0 | 8 | 11 |
| Gagnier | 2016 | 1.5 | 2 | 1 | 0 | 1 | 1 | 1 | 0 | 1 | 1 | 1 | 1 | 0 | 0 | 1 | 1 | 0 | 1 | 0 | 11.5 | 14.5 |
| Ottinger | 2017 | 1.5 | 3 | 1 | 0 | 1 | 0 | 1 | 0 | 0 | 1 | 1 | 1 | 1 | 1 | 1 | 1 | 0 | 0 | 0 | 10.5 | 14.5 |
| Stenquist | 2022 | 1.5 | 3 | 1 | 1 | 1 | 1 | 1 | 1 | 1 | 1 | 0 | 1 | 1 | 0 | 1 | 1 | 0 | 1 | 0 | 13.5 | 18.5 |
| Van Eaton | 2005 | 3 | 3 | 1 | 0 | 1 | 1 | 1 | 1 | 0 | 1 | 1 | 1 | 1 | 1 | 1 | 1 | 1 | 1 | 1 | 14 | 22 |
| Telem | 2011 | 2 | 3 | 1 | 1 | 1 | 1 | 1 | 1 | 0 | 1 | 1 | 1 | 1 | 0 | 1 | 1 | 0 | 0 | 0 | 14 | 18 |
| Britt | 2015 | 3 | 1 | 1 | 1 | 1 | 0 | 1 | 1 | 0 | 1 | 1 | 1 | 1 | 1 | 0 | 1 | 1 | 1 | 0 | 12 | 18 |

**Table S4.** Study interventions, outcomes, and results

| First author | Year | Interventions | Pre-intervention/ control | Outcome measures | Data collection methods | Population & sample size | Main results | *P* value |
| --- | --- | --- | --- | --- | --- | --- | --- | --- |
| Advani | 2015 | Handover tool:  Electronic, partially automated, linked with EPR (investigation results)  Process standardisation:  Handover policy  Mnemonic:  SBAR incorporated into handover tool  Staff education | Pre-intervention:  No standardised handover process (‘haphazard handovers’) | Process outcomes:  Compliance with BMA 2004 guidelines  Staff outcomes:  Satisfaction | Staff survey | Surgical residents  Pre: n=20  Post: n=13 | Self-reported improvement in adherence to guidelines:  Handover occurrence:  50🡪92.3-100%  Face-to-face handover: 50🡪100%  Designated space: 40🡪84.6%  Designated time: 40 🡪 100%  List available & accurate: 50-70🡪84.5-92.3%  Staff satisfaction with the handover process: 30%🡪91.6% | nr |
| Ahmed | 2012 | Handover tool:  Electronic, manually maintained, standalone document  Staff education:  40-min session: Didactic teaching & information pack | Pre-intervention:  No standardised handover process  Non-standardised electronic handover tools | Process outcomes:  Compliance with RCSE 2007 guidelines for handover content  Patient outcomes:  Patient safety incidence | Prospective audit of handover sheets  Collection of patient safety data | Acute surgical patients  Pre: n=137  Post: n=155 | Significant improvement in inclusion of 7 of 11 data points  No patient safety incidents reported pre- or post-intervention | <0.05 |
| Antonoff | 2012 | Process standardisation:  Handover policy  Staff education:  1-hour workshop:  Didactic teaching  Simulated handovers  Laminated pocket card | Pre-intervention:  No standardised handover process | Staff outcomes:  *Residents:*  Satisfaction with handover process  *Residents & nurses:*  Perceived safety of process  Perceived adequacy of information handed over  Perceived on-call resident knowledge of patients | Staff surveys:  Resident survey  Nursing survey (assessing physician handover) | Surgical residents  Pre: n= 37  Post: n= nr  Critical care and surgical ward nurses  Pre: n=46  Post: n=46 | Nonsignificant improvement in satisfaction with handover, perceptions of patient safety, adequacy of information provided in handover, and on-call resident knowledge of patients | nr |
| Bakti | 2017 | Handover tool:  Electronic handover database, partially automated (can be used to create customisable lists), standalone (MS Excel) | Pre-intervention:  Template-based handover system, no database  3 separate handover documents:   - Electronic - White board - Paper/hand-written | Process outcomes:  Completeness & adequacy of handover information as per BMA 2004 guidelines  Staff outcomes:  Perceived coordination and management of the surgical service | Prospective audit of handover sheets  Staff survey: Administered once in cycle 2 | Orthopaedic trauma patients:  Pre: n=301  Post: n=300  Trauma staff:  Pre: n=29  Post: n=29 | Significant improvement in inclusion of handover information (10 of 13 datapoints)  A significant improvement in perceived  ability to manage acute trauma referrals,  coordinate patients awaiting surgery, and  access previous handovers | <0.01  <0.001 |
| Bass | 2015 | Staff education:  Didactic teaching in specialty-specific common acute surgical cases & the importance of involving senior staff  Process standardisation:  Increased supervision by senior staff on call | Pre-intervention:  No education  Less senior staff involvement on call | Patient outcomes:  Change in diagnosis  Inadequate investigation  Inadequate treatment  Mortality  Process outcomes:  Occurrence of handover  Time to physician review | Retrospective audit of patient charts | Emergency urology patients:  Pre: n=47  Post: n=45 | Significant increase in the number of patients handed over to the oncoming urology team  Significant increase in the number of patients handed over to the urology registrar on call  No change in diagnosis after post-take ward round  Reduction in the number of patients with:  Inadequate investigation and Inadequate treatment (calculated by authors)  No change in time to SHO or Registrar review  No deaths | <0.05  <0.05  0.7  <0.05  <0.001  nr |
| Bethune | 2014 | Handover tool:  Paper document (handover sticker for chart)  Staff education:  Didactic teaching session for those using the sticker | Pre-intervention:  Non-standardised or absent handover notes | Patient outcomes:  Completion of weekend tasks  Process outcomes:  Documentation of a handover note in the chart | Audit of patient charts | Patients:  Pre: n=5  Post: nr | Tasks completed: 70🡪100%  Documented handovers: 25🡪72.5% | nr |
| Blower | 2014 | Handover tool:  Electronic, manually maintained, standalone document. More detailed, based on RCSE 2007 guidelines  Staff education:  Trainees received induction on use of the new tool | Pre-intervention:  Less detailed electronic handover template | Process outcomes:  Inclusion of recommended handover data points as per RCSE 2007 guidelines | Audit of handover sheets | Surgical patients:  Pre: n=118  Post: n=114 | Significant improvement in inclusion of 6/10 handover data points  Note: Documentation of outstanding tasks & frequency of review were poor both pre- and post-intervention | </= 0.01 |
| Bradley | 2014 | Process standardisation:  Handover policy  Handover tool:  Paper document, attached to front of patients notes for transfer.  Based on RCSE 2007 guidelines  Staff education:  Didactic teaching of FY1s on guidelines and handover tool | Pre-intervention:  Non-standardised or no formal handover process | Process outcomes:  Number of patients handed over from the CAU to the FY1 on the surgical ward  Quality of handover as determined by the RCSE 2007 recommended minimum dataset | Audit of handover sheets | Emergency surgical patients:  Pre: n=59  Post: n=56 | Number of patients handed over: 15🡪45%  Quality of patient handover: 0🡪100% of handovers including the recommended dataset | nr |
| Britt | 2015 | Staff education:  Didactic teaching &  simulated handovers | Control:  No education | Staff outcomes:  Handover performance as determined by a handover assessment tool developed by the research team | Blind rater assessment of videotaped handovers using the handover assessment tool | Surgical and paediatric interns:  Intervention: n=16  Control: n=16 | Trained interns performed significantly better than untrained interns on 5 of 6 elements, including: Organization, economy, confidence, order, and comprehension.  No change in professionalism (good scores in both the intervention and control groups) | <0.001  <0.01  <0.01  <0.05  <0.001 |
| Chopra | 2016 | Handover tool:  Electronic, manually maintained, bespoke handover software  Staff education:  Didactic teaching & provision of technical support to participants | Pre-intervention:  Electronic handover on MS Word | Staff outcomes:  Reported stress/difficulty associated with use of system  Perceived safety  Process outcomes:  Incorrect/missing patient information  Speed of use (minutes, mean (SD)) | Staff survey (developed through Delphi process) | Surgical residents:  Pre: n=25  Post: n=24 | Reduced number of staff reporting that creation of the handover was stressful (16-60🡪36-45.5%)  Increased number of staff reporting that creation of the handover sheet was difficult (0🡪9%)  Less staff with safety concerns re. the handover (60🡪32%)  Less staff reported missing/incorrect patient data (56🡪9%)  Less time spent creating post-take handover (27(7.5) vs 15(4)) | nr |
| Culwick | 2014 | Handover tool:  Electronic, manually maintained, unified handover document | Pre-intervention:  Multiple different non-standardised printed handover documents | Staff outcomes:  Satisfaction with the handover  Process outcomes:  Time taken to locate patients on the weekend list (seconds; mean (SD))  Time taken to compile weekend job list (range of minutes reported on Likert scale) | Staff survey  Timing of residents performing relevant activities | General surgery and urology residents  Survey:  Pre: n=14; post: n=14 | Satisfaction increased from 7.1🡪85.7%  Improvement in 7 of 8 self-reported measures of handover content  Reduced time taken to locate patients:  20.8 (6.2) vs 3.9 (2)  Reduced time taken to compile jobs:  20-40 vs 0-5 mins | nr |
| Dean | 2017 | Handover tool:  Paper handover document, based on RCSE 2007 guidelines  Process standardisation:  Highlighting patients who require urgent weekend senior review | Pre-intervention:  Electronic database with inadequate patient detail for weekend review | Patient outcomes:  Length of stay  30-day unplanned readmissions  Process outcomes:  Inclusion of recommended data points as per guidelines  System outcomes:  Weekend discharges  Estimated cost-savings | Audit of handover documents  Audit of weekend discharges, length of stay, and readmissions  Extrapolated cost savings based on reduced LOS | General surgery patients:  Total n=1426  Pre: n=nr  Post: n=nr | LOS for all patients whose stay included a weekend decreased by 1.54 days  LOS for emergency patients whose stay included a weekend decreased by 1.96 days  No change in readmissions  Increase in number of handovers with documented: Working diagnosis, management plan, and outstanding tasks  Increase in weekend discharges  Cost savings between £740 000 and £3.82 million. | 0.03  0.02  0.7  <0.0001  0.003  nr |
| Din | 2012 | Handover tool:  Paper handover document with traffic light system to flag patients for review  Staff education:  Didactic teaching  Advertising campaign  Process standardisation:  Traffic light system to flag patients for review incorporated into handover document | Pre-intervention:  No system for handover of inpatients at the weekend | Staff outcomes:  Confidence  Process outcomes:  Staff-report presence of a handover sheet & duration of weekend ward round  System outcomes:  Weekend discharges | Staff survey  Audit of weekend discharges | General surgical and vascular residents:  Pre: n=18  Post: n=18 | Confidence in dealing with patients over weekend (good/excellent) 22.2🡪72.2%  Handover sheet 35🡪85%  Ward rounds >3.5🡪<3 hours  Weekend discharges 5🡪20% | nr |
| Ellul | 2011 | Staff education:  Didactic teaching  Provision of RCSE guidelines  Reminders & feedback | Pre-intervention:  No teaching | Process outcomes:  Presence and completeness of morning & afternoon handovers (in the handover book) | Audit of handover book | Handovers (morning & afternoon):  Pre: n=30  Post: n=18 | Morning handover  77🡪94%  Afternoon handover  30🡪72% | nr |
| Ferran | 2008 | Handover tool:  Paper document based on RCSE 2007 guidelines | Pre-intervention:  Unstructured written handovers | Process outcomes:  Inclusion of recommended data points  Handover of tasks | Audit of handover proformas | Orthopaedic Patients:  Pre: n=48  Post: n=55 | Overall data handed over 72.6🡪93.2%  Handover of outstanding tasks 31.2🡪100% | <0.01  <0.01 |
| Gagnier | 2016 | Handover tool:  Electronic handover document, manually maintained, standalone  Staff education:  Briefing on the tool | Control:  Historical control group using less detailed proforma | Patient outcomes:  Adverse events  Length of stay (days; mean, 95% CI)  Process outcomes:  Time to prepare handovers  Method of handover | Staff survey  Audit of adverse events and length of stay | Orthopaedic patients:  Control: n=67  Intervention: n=60 | Reduction in adverse events per person  Any adverse events 59.7🡪51.7%  LOS 3.33 (1.86–4.79)🡪2.85 (1.44–4.26)  Increase in time to prepare handovers  Reduction in face-to-face & phone handovers, increase in email handovers | <0.1  nr  nr  nr  nr |
| Gibbons | 2015 | Process standardisation:  Introduction of a Friday handover meeting  Handover tool:  Electronic, manually maintained, standalone weekend handover document  Staff education:  Didactic teaching  Provision of feedback | Pre-intervention:  No formal Friday handover and no electronic patient list for weekend handover | Patient outcomes:  Readmission within 14 days  Number of ERT calls  LOS of discharged patients (mean)  System outcomes:  Weekend discharges | Audit | General surgical and vascular patients:  Pre: n=284  Post: n=310 | No readmissions in any period  Reduction in mean LOS from 13 to 5.4 days  Reduction in total number of ERT calls for the month from 7 to 4  12 to 4 from cycle 2-3  Increase in weekend discharges (10.6🡪14.8%) | <0.05  nr  <0.05  <0.05 |
| Jacob | 2021 | Handover tool:  Electronic, manually maintained document using NerveCentreTM software  Provision of iPads for handover (linked with EPR)  Staff education:  Provision of training documents | Pre-intervention:  Electronic handover template using MS Word and printed patient lists | Staff outcomes:  Perceptions of efficiency of ward rounds, information governance,  patient care  Process outcomes (staff reported):  Time taken to prepare the handover  Transfer of information to nursing staff  Availability of information | Staff survey | Surgical residents:  Pre: n=37  Post: n=23 | Improved ward round efficiency (41🡪82%)  Improved information governance (19.5🡪82%)  Reduction in misplaced patient lists (nr)  Reduction in number of doctors accidentally taking patient list home (nr)  Improved perception of patient care (30🡪48%)  Reduction in time taken to prepare the handover  Improver transfer of information to nursing staff (13.9🡪69.6%)  Improved availability of information at the bedside | 0.002  <0.001  <0.006  <0.001  nr  0.012  <0.001  </=0.012 |
| Jardine | 2014 | Handover tool:  Electronic, partially automated handover document, integrated with EPR | Pre-intervention:  Handwritten paper list of weekend jobs left for weekend on-call staff to collect | Staff outcomes:  Perceptions of patient safety  Process outcomes:  Quality of handover | Audit of handovers using a scoring system  Staff survey | General internal medicine and general surgical patients (n=nr)  FY1 doctors | Improved quality of weekend handover (76🡪93%)  Improved staff perceptions of format of handover and impact on patient safety | <0.01  nr |
| Jones | 2015 | Handover tool:  Paper document, based on RCSE 2007 guidelines | Pre-intervention:  No standardised handover template | 1. Process outcomes:  Inclusion of recommended data points (RCSE 2007)  Duration of handover | Audit of handovers | General surgical patients:  Pre: n=64  Post: n=53 | The documentation of 9 of 13 data points increased significantly  No difference in duration of handover (25 vs 27 minutes) | <0.05 |
| Khanna | 2015 | Handover tool:  Mobile phone application (WhatsApp)  Staff education:  Didactic teaching and training on use | Pre-intervention:  No standardised handover process  (combination of written handovers and use of a paging system) | Staff outcomes:  Surgical residents awareness of patient-related information  Process outcomes:  Duration of handover | Staff survey  Measurement of handover duration | Orthopaedic residents:  Pre: n=8  Post: n=8  Handovers:  n=nr | Improvement in the awareness of patient diagnosis  No improvement in awareness of patient management  Reduced time of written handovers in the morning (25🡪14 mins) | 0.024  0.145  0.003 |
| Khoury | 2018 | Handover tool:  Electronic patient census printed from EPR, no handover details | Pre-intervention:  Non standardised  electronic handover sheets | Patient outcomes:  Occurrence of postop day 1 review over the weekend | Audit of patient notes | Orthopaedic patients (postoperative):  Pre: n=26  Post: n=48 | Postop reviews increased from 54🡪96% | nr |
| Krishna | 2018 | Process standardisation:  Introduction of a twice daily multidisciplinary handover meeting (‘huddle’) | Pre-intervention:  No standardised handover | Patient outcomes:  Number of patients transferred after hours to another hospital | Numbers of transfers | Total elective surgical patients transferred:  Pre: n= 71  Post: n= 69 | There were no significant differences in the proportion of after-hours transfers (48 vs 45)  Significant increase in the number of patients being transferred for further radiological investigations  Significant increase in the proportion of transfers under the presumed diagnosis of VTE | P=0.76  0.033  0.0023 |
| Krushelnytskyy | 2022 | Handover tool:  Electronic handover document updated to include neurological exam integrated with EPR | Pre-intervention:  Electronic handover without SOAP format or neuro exam | Process outcomes:  Time taken to write patient progress notes (seconds, mean (SD)) | Measurement of time taken to write handover | Neurosurgical patient progress notes:  Pre: n=121  Post: n=69 | Reduced time taken to write notes (120.3(16.8)🡪37.9s(12.4)) | 6.66 × 10–11 |
| Law* | 2022 | Handover tool:  Flowchart to structure handover  Process standardisation:  Implementation of local guidelines  Mnemonic: ISBAR  Staff education.  Advertising campaign | Pre-intervention:  Non-standardised and unstructured handover | Process outcomes:  Handover: delay, duration, attendance, content | Direct observation of handovers | Handovers:  Pre: n=8  Post: n=8  Medical and surgical patients:  Pre: n=50  Post: n=46 | Improvements in 6 of 10 variables for content & structure of handover  Improved attendee lateness & attendance  Reduced average handover delays (9🡪2mins) and duration (17🡪12 min) | nr |
| Lee | 2014 | Handover tool:  Paper, hand-written organ-based handover checklist  Process standardisation:  Mandatory morning handover | Control:  Historical control group | Patient outcomes:  Complication rates  Mortality rates  Median (IQR) overall LOS  Median ICU LOS | Data collection by trauma case managers | Trauma surgical patients:  Control: n=824  Intervention: n=798 | No difference in complications or mortality rate  Reduction in:  Overall LOS 2(1-5)🡪2(1-4)  ICU LOS (2🡪1 days) | 0.86  0.12  <0.001  0.007 |
| Maroo | 2017 | Handover tool  Electronic, manually maintained document (MS Word)  Staff education  On tool usage | Pre-intervention  No formal handover process  Paper handover sheets | Staff outcomes:  Perceived: Safety, handover quality out of 10, legibility | Staff survey  Staff focus group | Orthopaedic residents:  Pre: n=8  Post: n=8 | Handover rating 3.4🡪8  Handover contained enough information to safely review a patient (75🡪100%)  Legible (0🡪100%)  All jobs are necessary (25🡪75%)  Helps prioritise jobs (50🡪25%) | nr |
| Ottinger | 2017 | Staff education  Didactic teaching | Pre-intervention:  No education | Process outcomes:  Handover quality | Observation & assessment using an evaluation tool | Patient handovers:  Pre: n=50  Post: n=250 | Significant improvement in inclusion of handover information and communication skills of the residents | <0.01 |
| Piscioneri | 2011 | Process standardisation:  Handover meeting  Handover tool:  Electronic, manually maintained document  Staff education | Pre-intervention:  Informal verbal handover | Process outcomes:  Tertiary survey completion rates (trauma documentation)  Time to completion of survey | Audit of tertiary survey completion rates | nr | Documentation completion rate  29.6🡪86.1%  Time to completion 30.6🡪32.8hours | nr |
| Ramsay | 2018 | Handover tool:  Paper handover,  linked with nursing handover  Mnemonic: SBAR  Staff education: Didactic teaching  Posters | Pre-intervention:  Informal handover | Patient outcomes:  Staff-reported frequency of missed tasks | Staff survey | FY1 doctors:  Pre: n=15  Post: n=10 | Reduction in 7 of 9 missed tasks (antibiotic levels/prescriptions, blood tests, medication doses, patient paperwork, referrals to other specialties, senior review, repeat troponin) | nr |
| Raptis | 2009 | Handover tool:  Electronic, manually maintained, handover document | Pre-intervention:  Paper handover proforma (handwritten fields) | Process outcomes:  Handover quality (completed handovers). Proportion of patients handed over | Audit of handover documents | Surgical and medical patients:  Pre: n=773  Post: n=872 | Increased inclusion of information in the electronic handover document  13(13-29) Vs 17(9-40) patients handed over | </=0.0002  0.0001 |
| Raval | 2015 | Handover tool:  Electronic handover document, integrated with EPR, partially automated | Pre-intervention:  Electronic, manual handover (MS Access Database) with some handwritten fields | Process outcomes:  Errors in handover document  List maintenance time (mins)  Patient outcomes:  Codes outside of the intensive care unit  Serious safety events  ADEs related to prescribing of antibiotics  GI surgery infection rate  Readmission rate  Staff outcomes: Perceived efficiency, accuracy, safety of handover | Staff survey  Audit of patient outcomes  Audit of handover documents | Surgical staff:  Pre: n=21  Post: n=29  Handovers:  Pre: n=5  Post: n=5  Surgical patients:  nr | List maintenance time: 155.75 (30–360) 🡪 112.59(20–420)  Perceived: Efficiency: 28.6🡪89.7%  Accuracy: 33.3🡪79.4%  Safety: 42.8🡪79.4%  No change in: Codes outside of ICU, serious safety events, ADEs, readmission rates  **GI surgical infection rates**  **6.3🡪4%**  Errors in handover document (19%🡪0%) | 0.16  <0.01  0.01  0.01  **<0.01** |
| Ryan | 2010 | Handover tool:  Electronic, manually maintained, handover document  Process standardisation:  Verbal handover meeting | Pre-intervention:  Written paper handover | Patient outcomes:  Median LOS  Time to first intervention (CT): Mean +/- SEM (hours) | Audit of LOS | Emergency general surgical patients:  Pre: n=47  Post: n=41 | LOS 5 🡪 4 days  No difference in time to CT (21.2+/-10.3 versus 28.2 +/-7.8) | 0.047  0.059 |
| Sadri | 2013 | Handover tool:  Paper document (based on RCSE 2007 guidelines)  Mnemonic:  ABCD (based on ATLS principles)  Process standardisation:  Policy | Pre-intervention:  No standardised handover proforma | Process outcomes:  Location of handover  Senior supervision  Number of patients handed over  Inclusion of all patient information  Number of bleeps during handover  Duration of handover (mins)  Staff outcomes: Staff overtime (mins) | Audit of handover | Handovers:  Pre: n=21  Post: n=21 | Handover in non-clinical environment (48🡪76%)  Senior supervision (23🡪85.7%)  All patients handed over (69🡪100%)  Complete patient information (71.4🡪90.5%)  No change in bleeps during handover (61.9🡪66.7 %)  No change to handover duration (14🡪17)  Reduced overtime (16.9🡪45.7) | 0.3  nr  nr  <0.05  0.29  0.14  <0.05 |
| Stenquist | 2022 | Handover tool:  Electronic, incorporated into EPR, manually maintained, document  Mnemonic:  Modified I-PASS – ‘OrthoPass’  Staff education | Pre-intervention:  Non-standardised Paper based system | Process outcomes:  Quality of handover  Patient outcomes:  Adverse clinical outcomes  Readmissions | Audit of handover  Collection of adverse event data  Staff survey | Orthopaedic patients:  Pre: n=1012  Post: n=972  Handovers:  Pre: n=203  Post: n=100  Staff:  Pre: n=74  Post: n=74 | Handover quality: Improvement  in 8 of 9 targeted quality elements (illness severity, medical history, action list, situational awareness, contingencies, anticoagulation plan, and antibiotic plan)  No differences in adverse events or readmissions | </=0.002 |
| Telem | 2011 | Staff education:  SBAR training incorporated into surgical curriculum (2.5 hours), Simulated patient handovers, Lecture & video  Mnemonic:  SBAR as above | Control:  No training | Patient outcomes:  Adverse events  Process outcomes:  Resident erroneous order entries | Audit of electronic order entries & morbidity and mortality surgical database | General surgical (intervention; n=38) and subspecialty (control; n=20) residents  Order entries:  Pre: n=6360  Post: n=6873 | Significant reduction in erroneous order entries in the intervention group (14.5🡪12.2%)  No change in the control group  No difference in sentinel events | 0.003 |
| Van Eaton  Van Eaton | 2005  2010 | Handover tool:  Electronic document, incorporated into EPR, automatically downloads labs and vital signs. ‘UWCores’  Staff education:  6-week pilot with system | Control:  Usual practice (no standardised handover template) | Patient outcomes:  Continuity of care (staff-reported number of patients missed on ward rounds)  Resident reported incidents & deviations in expected care  Perceived causes of: Deviations, medical errors, ADEs  Process outcomes:  Self-reported workflow efficiency – Duration of ward round, time spent at bedside pre-rounds | Telephone staff surveys  Electronic staff surveys  Review of hospital quality assurance database.  Review of reported medical errors by blinded researcher | Patients:  Intervention: n=8018  Control: n=7569  Resident teams:  Intervention: n=7  Control: n=7 | Number of patients missed on ward rounds reduced from 5🡪2.5 patients/team/month  Reduced mean pre-rounding time spent hand-copying vital signs and labs (24🡪12%)  Reduced duration of ward round by 1.5 minutes per patient  No change in:  Total reported incidents  Total reported errors  Reported overnight medical errors  Adverse drug events | 0.0001  <0.0001  0.0006  0.66  0.86  0.66  0.7 |
| Wayne | 2008 | Handover tool:  Electronic document, manually maintained, standalone, handover document (MS Excel) | Pre-intervention:  Non-standardised electronic handover (MS Excel) – different format used by each team | Staff outcomes:  Perceived: Accuracy & completeness of information, clarity of time of transfer of patient responsibility, appropriateness of tasks handed over  (Lower scores = better result) | Staff surveys (telephone & electronic) | Surgical residents (non-ICU):  Pre: n=60  Post: n=101 | Non-ICU perceived accuracy (2.9🡪2.1)  ICU perceived accuracy (3🡪2.1)  Non-ICU perceived completeness (3.55 to 2.5). ICU (1.8🡪2.2)  Time of transfer clarity for non-ICU night shifts (4.6🡪3.8)  Non-ICU task appropriateness (1.1🡪0.6) | 0.006  0.142  0.005  0.312  0.008  0.049 |
| White-Gibson | 2018 | Handover tool:  Electronic patient list linked with EPR  Process standardisation:  Formal face to face handover  ‘Red flag’ system incorporated into patient list  Staff education: Teaching session | Pre-intervention:  Informal handover & no red flag system | Process outcomes:  Mean number of weekend clinical events reported back to the primary team on Monday morning | Audit | Weekend handovers:  Pre: n=6  Post: n=6  Surgical patients:  n=nr | Mean clinical events reported per weekend 3.17± 0.6 🡪 7.83± 1.2 | 0.007 |
| Wohlauer | 2011 | Handover tool:  Electronic handover document, linked with EPR, partially automated | Pre-intervention:  Electronic standalone handover (MS Excel) | Process outcomes:  Mean time pre-rounding  Patient outcomes:  Missed patients on rounds | Staff surveys | Medical *and* surgical residents  Pre: n=168; Post: n=83 **(Surgical Residents** Pre: n=52; post: n=37) | Surgical resident pre-rounding time 40.1🡪31.2  No patients missed on rounds 56.5🡪69.9% | 0.09  0.001 |
| Wolinska | 2021 | Handover tool:  Electronic handover document, linked with EPR, partially automated  Staff education:  2-hour I-PASS workshop, ongoing coaching  Mnemonic: I-PASS, used to structure handovers | Pre-intervention:  Manually maintained excel handover document. Unstructured verbal handover & no training | Staff outcomes:  Satisfaction  Process outcomes:  Inclusion of I-PASS data points in verbal & written handover  Duration of handover (mins) | Observation of evening handovers  Assessment of written & verbal handovers using I-PASS checklist  Staff survey | Verbal handovers:  Pre: n=25  Post: n=25  Written handovers:  Pre: n=24  Post: n=25  Surgical residents:  n=nr | Improved staff satisfaction in all survey areas  Improvement in inclusion of 12 elements of the I-PASS checklist for verbal handover  Improvement in inclusion of 9 elements of the checklist for written handover  Increased duration of handover (20(10–30)🡪25(15–40) | </=0.001  </=0.001  <0.05  <0.01 |

*EPR, Electronic patient record; I/SBAR, identity, situation, background, assessment, recommendation; BMA, British Medical Association; nr, not reported; RCSE, Royal College of Surgeons in England; FY, Foundation Year; CAU, combined assessment unit; MS, Microsoft; SD, standard deviation; LOS, length of stay; ERT, Emergency response team; SOAP, subjective, objective, assessment, plan; IQR, interquartile range; ICU, intensive care unit; GI, gastrointestinal; ADE, adverse drug event; CT, computed tomography; SEM, standard error of the mean; ATLS, advanced trauma life support; IPASS, , Illness severity, patient summary, action list, situation awareness and contingency planning, synthesis by receiver

**Table S5a.** Patient outcomes

|  | **Studies with significant improvement (n)** | **Sample size**  **Control** | **Sample size Intervention** | **Improvement seen but no *p* value given** |
| --- | --- | --- | --- | --- |
| Adverse events (n=7)  ^3-9^ | 1^6^ | nr | nr | 1^4^ |
| Length of stay (n=5)  ^4,5,10-12^ | 4^5,10-12^ | 1635 | 1629 | 1^4^ |
| Readmission rate (n=4)  ^6,7,10,11^ | 0 |  |  | 0 |
| Review on ward round (n=3)  ^13-15^ | 2 ^13,14^ | 8018 | 7569 | 1^15^ |
| Task completion rate (n=2)  ^16,17^ | 0 |  |  | 2^16,17^ |
| Reason for transfer (n=1)  ^18^ | 1^18^ | 71 | 69 | 0 |
| Mortality (n=2)  ^5,19^ | 0 |  |  | 0 |
| ERT calls/codes (n=2)  ^6,11^ | 1^11^ | 284 | 310 | 0 |
| Inadequate treatment (n=1)  ^19^ | 1^19^ | 47 | 45 | 1^19^ |
| Inadequate investigation (n=1)  ^19^ | 1^19^ | 47 | 45 | 1^19^ |
| Change in diagnosis after senior review (n=1)  ^16^ | 0 |  |  | 0 |
| Time to CT scan (n=1)  ^12^ | 0 |  |  | 0 |

**nr, not reported; ERT, emergency response team; CT, computed tomography*

**Table S5b.** Process outcomes

|  | **Studies with significant changes (n)** | **Changes seen but no *p* value given (n)** |
| --- | --- | --- |
| **Pre-handover** | | |
| Time taken to prepare handover (n=4)^4,6,20,21^ | 1^21^ | 1^20^ |
| Duration of pre-round (n=2)^13,14^ | 1^13^ | 0 |
| Time taken to compile weekend job list (n=1)^22^ | 0 | 1^22^ |
| Time taken to locate patients on the list (n=1)^22^ | 0 | 1^22^ |
| **Handover logistics** | | |
| Duration of handover (n=5)^23-27^ | 1^26^ (reduced)  1^23^ (increased) | 1^27^ (reduced) |
| Handover occurrence (n=3)^19,28,29^ | 1^19^ | 2^28,29^ |
| Method of handover (n=2)^4,28^ | 0 | 2^4,28^ |
| Location of handover (n=2)^25,28^ | 0 | 1^28^ |
| Available patient list (n=2)^28,30^ | 0 | 2^28,30^ |
| Handover leader/supervision (n=1)^25^ | 0 | 1^25^ |
| Designated time for handover (n=1)^28^ | 0 | 1^28^ |
| Handover delay (n=1)^27^ | 0 | 1^27^ |
| Handover attendance (n=1)^27^ | 0 | 1^27^ |
| Bleeps during handover (n=1)^25^ | 0 | 0 |
| **Handover content** | | |
| Written handover information (n=13)^3,7,10,23-25,29,31-36^ | 11^3,7,10,23-25,31-35^ | 1^29,36^ |
| Verbal handover information (n=3)^23,27,37^ | 2^23,37^ | 1^27^ |
| Errors in handover document (n=2)^6,20^ | 0 | 2^6,20^ |
| Number of patients handed over (n=2)^25,32^ | 1^32^ | 1^25^ |
| Number of clinical events reported (n=1)^38^ | 1^38^ | 0 |
| Handover of tasks (n=1)^35^ | 1^35^ | 0 |
| Transfer of information to nursing staff (n=1)^21^ | 1^21^ | 0 |
| **Post-handover** | | |
| Duration of ward round (n=2) ^13,30^ | 1^13^ | 1^30^ |
| Completion of documentation (n=2) ^16,39^ | 0 | 2 ^16,39^ |
| Time to patient review (n=1)^19^ | 0 | 0 |
| Time taken to complete documentation (n=2)^39,40^ | 1^40^ (reduced) | 1^39^ (increased) |
| Erroneous order entries (n=1)^8^ | 1^8^ | 0 |
| Availability of information at the bedside (n=1)^21^ | 1^21^ | 0 |

**Table S5c.** Staff outcomes

|  | **Studies with significant improvement (n)** | **Sample size**  **Control** | **Sample size Intervention** | **Improvement seen but no *p* value given (n)** |
| --- | --- | --- | --- | --- |
| Perceived safety of the handover process (n=6)^6,20,21,33,41,42^ | 1^6^ | 21 | 29 | 4^20,21,33,41^ |
| Perceived handover quality  (n=5)^6,7,41-43^ | 3^6,7,43^ | 155 | 204 | 1^41^ |
| Staff satisfaction (n=4)^22,23,28,42^ | 1^23^ | nr | nr | 2^22,28^ |
| Staff knowledge of patients (n=2)^26,42^ | 1^26^ | 8 | 8 | 0 |
| Perceived efficiency of the process (n=1)^6^ | 1^6^ | 21 | 29 | 0 |
| Perceived impact on coordination of the surgical service (n=1)^31^ | 1^31^ | 29 | 29 | 0 |
| Stress/difficulty associated with the handover process (n=1)^20^ | 0 |  |  | 0 |
| Confidence in dealing with patients on call (n=1)^30^ | 0 |  |  | 1^30^ |
| Perceived ward round efficiency (n=1)^21^ | 1^21^ | 37 | 23 | 0 |
| Perceived information governance (n=1) ^21^ | 1^21^ | 37 | 23 | 0 |
| Perceived legibility of handover (n=1)^41^ | 0 |  |  | 1^41^ |
| Staff overtime (n=1)^25^ | 1^25^ | nr | nr | 0 |
| Clarity of transfer of patient responsibility (n=1)^43^ | 1^43^ | 60 | 101 | 0 |
| Handover performance on assessment (n=1)^44^ | 1^44^ | 16 | 16 | 0 |

**REFERENCES**

1. Riesenberg LA, Leitzsch J, Massucci JL, Jaeger J, Rosenfeld JC, Patow C, et al. Residents' and attending physicians' handoffs: a systematic review of the literature. Acad Med. 2009;84(12):1775-87.

2. Downs SH, Black N. The feasibility of creating a checklist for the assessment of the methodological quality both of randomised and non-randomised studies of health care interventions. Journal of Epidemiology & Community Health. 1998;52(6):377-84.

3. Ahmed J, Mehmood S, Rehman S, Ilyas C, Khan LU. Impact of a structured template and staff training on compliance and quality of clinical handover. Int J Surg. 2012;10(9):571-4.

4. Gagnier JJ, Derosier JM, Maratt JD, Hake ME, Bagian JP. Development, implementation and evaluation of a patient handoff tool to improve safety in orthopaedic surgery. Int J Qual Health Care. 2016;28(3):363-70.

5. Lee JC, Horst M, Rogers A, Rogers FB, Wu D, Evans T, et al. Checklist-styled daily sign-out rounds improve hospital throughput in a major trauma center. The American surgeon. 2014;80(5):434-40.

6. Raval MV, Rust L, Thakkar RK, Kurtovic KJ, Nwomeh BC, Besner GE, et al. Development and implementation of an electronic health record generated surgical handoff and rounding tool. J Med Syst. 2015;39(2):8.

7. Stenquist DS, Yeung CM, Szapary HJ, Rossi L, Chen AF, Harris MB. Sustained Improvement in Quality of Patient Handoffs After Orthopaedic Surgery I-PASS Intervention. J Am Acad Orthop Surg Glob Res Rev. 2022;6(9).

8. Telem DA, Buch KE, Ellis S, Coakley B, Divino CM. Integration of a formalized handoff system into the surgical curriculum: resident perspectives and early results. Arch Surg. 2011;146(1):89-93.

9. Van Eaton EG, McDonough K, Lober WB, Johnson EA, Pellegrini CA, Horvath KD. Safety of using a computerized rounding and sign-out system to reduce resident duty hours. Academic Medicine. 2010;85(7):1189-95.

10. Dean J, Phillips G, Turner W, Refson J. Demonstrating Improved Surgical Communication and HAndoveR Generates Earlier Discharges (DISCHARGED). J Patient Saf. 2018;14(3):e39-e44.

11. Gibbons JP, Nugent E, Tierney S, Kavanagh D. Implementation of a surgical handover tool in a busy tertiary referral centre: a complete audit cycle. Ir J Med Sci. 2016;185(1):225-9.

12. Ryan S, O'Riordan JM, Tierney S, Conlon KC, Ridgway PF. Impact of a new electronic handover system in surgery. Int J Surg. 2011;9(3):217-20.

13. Van Eaton EG, Horvath KD, Lober WB, Rossini AJ, Pellegrini CA. A randomized, controlled trial evaluating the impact of a computerized rounding and sign-out system on continuity of care and resident work hours. Journal of the American College of Surgeons. 2005;200(4):538‐45.

14. Wohlauer MV, Rove KO, Pshak TJ, Raeburn CD, Moore EE, Chenoweth C, et al. The computerized rounding report: implementation of a model system to support transitions of care. J Surg Res. 2012;172(1):11-7.

15. Khoury A, Jones M, Buckle C, Williamson M, Slater G. Improving weekend review for trauma and elective orthopaedic patients in the post-operative period. International Journal of Health Governance. 2018;23(4):264-8.

16. Bethune R, Campbell K, Rose A, Wassall R, Price C, Siese T, et al. Improving weekend handover between junior doctors on medical and surgical wards. BMJ Qual Improv Rep. 2014;2(2).

17. Ramsay N, Maresca G, Tully V, Campbell K. Does a multidisciplinary approach have a beneficial effect on the development of a structured patient handover process between acute surgical wards in one of Scotland's largest teaching hospitals? BMJ Open Qual. 2018;7(3):e000154.

18. Krishna S, Bae BJY, Coomarasamy C, Stapelberg F, Morton RP. The impact of introducing handovers on afterhours patient transfers in an elective surgery centre. Ambulatory Surgery. 2018;24(3):66-70.

19. Bass E, Patel S. Improving the handover and care of acute urological admissions. BMJ Qual Improv Rep. 2015;4(1).

20. Chopra S, Hachach-Haram N, Baird DL, Elliott K, Lykostratis H, Renton S, et al. Integrated Patient Coordination System (IntPaCS): a bespoke tool for surgical patient management. Postgrad Med J. 2016;92(1086):208-16.

21. Jacob N, Chaudhary O, Darwish NM, Vijay V, Pardoe H. E-Handover in Surgery Improves Clinical Efficiency and Adherence to COVID-19 Infection Control Measures. Cureus. 2021;13(3):e13967.

22. Culwick C, Devine C, Coombs C. Improving surgical weekend handover. BMJ Qual Improv Rep. 2014;3(1).

23. Wolinska JM, Lapidus-Krol E, Fallon EM, Kolivoshka Y, Fecteau A. I-PASS enhances effectiveness and accuracy of hand-off for pediatric general surgery patients. Journal of pediatric surgery. 2022;57(4):598-603.

24. Jones HG, Watt B, Lewis L, Chaku S. Structured Handover in General Surgery: An Audit of Current Practice. J Patient Saf. 2019;15(1):7-10.

25. Sadri A, Dacombe P, Ieong E, Daurka J, De Souza B. Handover in plastic surgical practice: The ABCD principle. European Journal of Plastic Surgery. 2014;37(1):37-42.

26. Khanna V, Sambandam SN, Gul A, Mounasamy V. "WhatsApp"ening in orthopedic care: a concise report from a 300-bedded tertiary care teaching center. European journal of orthopaedic surgery & traumatology : orthopedie traumatologie. 2015;25(5):821-6.

27. Law SJ, Seal ST, Cheepvasarach C. Improving the Medical and Surgical Out-of-Hours Handover at a Hospital in Regional New South Wales, Australia. Cureus. 2022;14(8):e27613.

28. Advani R, Stobbs NM, Killick N, Kumar BN. "Safe handover saves lives": Results from clinical audit. Clinical Governance. 2015;20(1):21-32.

29. Bradley A. Improving the quality of patient handover on a surgical ward. BMJ quality improvement reports. 2014;3(1).

30. Din N, Ghaderi S, O'Connell R, Johnson T. Strengthening surgical handover: Developing and evaluating the effectiveness of a handover tool to improve patient safety. BMJ Qual Improv Rep. 2012;1(1).

31. Bakti NI, Williamson M, Sehjal R, Thilagarajah M. The use of microsoft excel as an electronic database for handover and coordination of patients with trauma in a district general hospital. BMJ Innovations. 2017;3(3):130-6.

32. Raptis DA, Fernandes C, Chua W, Boulos PB. Electronic software significantly improves quality of handover in a London teaching hospital. Health informatics journal. 2009;15(3):191-8.

33. Jardine AG, Page T, Bethune R, Mourant P, Deol P, Bowden C, et al. Bring on the weekend - Improving the quality of junior doctor weekend handover. BMJ Qual Improv Rep. 2014;2(2).

34. Blower EL, MacCarrick T, Forster H, Sutton PA, Vimalachandran D. Implementation of a new tool to improve the efficacy and safety of surgical handovers. Int J Surg. 2015;13:189-92.

35. Ferran NA, Metcalfe AJ, O'Doherty D. Standardised proformas improve patient handover: audit of trauma handover practice. Patient Safety in Surgery. 2008;2(1):1-5.

36. Ellul D, Robson AK. Audit of handover in an ENT unit. The Journal of laryngology and otology. 2011;125(9):924-7.

37. Ottinger ME, Monaghan SF, Gregg SC, Stephen AH, Connolly MD, Harrington DT, et al. Trauma morning report is the ideal environment to teach and evaluate resident communication and sign-outs in the 80 hour work week. Injury. 2017;48(9):2003-9.

38. White-Gibson A, Manole C, Kearney D, Kavanagh D. Weekend surgical handover enhances early management of acute clinical changes using a red flag system. Ir J Med Sci. 2018;187(2):297-300.

39. Piscioneri F, Chong GC. Surgical handover in a tertiary hospital: a working model. Aust Health Rev. 2011;35(1):14-7.

40. Krushelnytskyy MD, Youngblood MW, Lesniak MS, Kemeny HR, Fernandez LG, Burdett KLB, et al. Optimizing the patient handoff and progress note documentation efficiency in the EPIC EMR system within a neurosurgery residency: A quality improvement initiative. Journal of clinical neuroscience : official journal of the Neurosurgical Society of Australasia. 2022;105:86-90.

41. Maroo S, Raj D. Introducing a New Junior Doctor Electronic Weekend Handover on an Orthopaedic Ward. BMJ quality improvement reports. 2017;6(1).

42. Antonoff MB, Berdan EA, Kirchner VA, Krosch TC, Holley CT, Maddaus MA, et al. Who's covering our loved ones: surprising barriers in the sign-out process. American journal of surgery. 2013;205(1):77-84.

43. Wayne JD, Tyagi R, Reinhardt G, Rooney D, Makoul G, Chopra S, et al. Simple standardized patient handoff system that increases accuracy and completeness. J Surg Educ. 2008;65(6):476-85.

44. Britt RC, Ramirez DE, Anderson-Montoya BL, Scerbo MW. Resident handoff training: initial evaluation of a novel method. J Healthc Qual. 2015;37(1):75-80.
